# Genome-wide identification, expression and salt stress tolerance analysis of the GRAS transcription factor family in *Betula platyphylla*

**DOI:** 10.3389/fpls.2022.1022076

**Published:** 2022-10-24

**Authors:** Zihang He, Zengzhi Tian, Qun Zhang, Zhibo Wang, Ruikun Huang, Xin Xu, Yucheng Wang, Xiaoyu Ji

**Affiliations:** ^1^ College of Forestry, Shenyang Agricultural University, Shenyang, Liaoning, China; ^2^ The Key Laboratory of Forest Tree Genetics, Breeding and Cultivation of Liaoning Province, Shenyang Agricultural University, Shenyang, Liaoning, China; ^3^ State Key Laboratory of Tree Genetics and Breeding, Northeast Forestry University, Harbin, China

**Keywords:** GRAS transcription factors, *Betula platyphylla*, genome-wide analysis, gene expression, salt stress tolerance

## Abstract

The *GRAS* gene family is a plant-specific family of transcription factors and play a vital role in many plant growth processes and abiotic stress responses. Nevertheless, the functions of the *GRAS* gene family in woody plants, especially in *Betula platyphylla* (birch), are hardly known. In this study, we performed a genome-wide analysis of 40 *BpGRAS* genes (*BpGRASs*) and identified typical GRAS domains of most *BpGRASs*. The *BpGRASs* were unevenly distributed on 14 chromosomes of birch and the phylogenetic analysis of six species facilitated the clustering of 265 GRAS proteins into 17 subfamilies. We observed that closely related GRAS homologs had similar conserved motifs according to motif analysis. Besides, an analysis of the expression patterns of 26 *BpGRASs* showed that most *BpGRASs* were highly expressed in the leaves and responded to salt stress. Six *BpGRASs* were selected for *cis*-acting element analysis because of their significant upregulation under salt treatment, indicating that many elements were involved in the response to abiotic stress. This result further confirmed that these *BpGRASs* might participate in response to abiotic stress. Transiently transfected birch plants with transiently overexpressed 6 *BpGRASs* and RNAi-silenced 6 *BpGRASs* were generated for gain- and loss-of-function analysis, respectively. In addition, overexpression of *BpGRAS34* showed phenotype resistant to salt stress, decreased the cell death and enhanced the reactive oxygen species (ROS) scavenging capabilities and proline content under salt treatment, consistent with the results in transiently transformed birch plants. This study is a systematic analysis of the *GRAS* gene family in birch plants, and the results provide insight into the molecular mechanism of the *GRAS* gene family responding to abiotic stress in birch plants.

## Introduction

Plants have to deal with various abiotic stresses in order to survive in a natural environment. As it is one of the most widely distributed adversity stresses, salinity can cause oxidative, ionic, and osmotic effects, thereby affecting the survival and growth of plants. Plants need to be able to tolerate and respond to the harm under salt stress conditions, facilitate ion regulation and osmotic equilibrium, improve anti-oxidase activity, and reduce harm. Transcription factors (TFs) are a class of proteins that can bind to certain deoxyribonucleic acid (DNA) sequences and control DNA transcription (Latchman, 1997), and usually play an important role in the generation of adaptive responses. So far, plant TFs from various species, such as NAC, bZIP, WRKY, and MYC/MYB, have been reported to be involved in abiotic stress responses. *ATAF1* is heterotopic and improves salt tolerance in *Oryza sativa* (rice) (Liu et al., 2016). The expression of *EsNAC1* is induced in response to salt stress in *Arabidopsis thaliana* (Arabidopsis) (Liu et al., 2018). *SlAREB1* and *SlAREB2*, from the *ABF* subfamily of *bZIP*, are involved in abscisic acid (ABA) pathways and the response to abiotic stresses such as salt and drought stress (Orellana et al., 2010). In woody species, *FcWRKY40* has been induced by ABA and salt treatment, and actively regulates salt tolerance by activating the salt-over-sensitivity (SOS) pathway in *Fortunella crassifolia* (Dai et al., 2018). ThCRF1 can enhance trehalose and proline biosynthesis and increase the reactive oxygen species (ROS) scavenging capability, thereby improving salt stress tolerance (Qin et al., 2017).

The *GRAS* gene family is a plant-specific TF family (Benfey et al., 1993), and was named after the three initially identified family members, i.e., Gibberellin acid insensitive (GAI), Repressor of GA1 (RGA), and Scarecrow (SCR) (Pysh et al., 1999). GRAS proteins have a variable N-terminal region and a highly conserved C-terminal region (Jaiswal et al., 2022), known as the GRAS domain, which includes 5 sequence motifs: leucine heptad repeat I (LHRI), leucine heptad repeat II (LHRII), VHIID, PFYRE, and SAW (Pysh et al., 1999). To date, GRAS TF family members of many species have been identified from the genome data of plants, such as Arabidopsis (Tian et al., 2004), rice (Tian et al., 2004), *Zea mays* (Guo et al., 2017), *Gossypium hirsutum* (Zhang et al., 2018), *Juglans regia* (Quan et al., 2019), and *Glycine max* (Wang et al., 2020). GRAS TF family members have huge genes, and these members have diverse structures. The differences in the sequence, structure, and phylogenetic relationship are considered to be major factors affecting the classification of GRAS family members. The *GRAS* gene family contains many subfamilies that exhibit not only great similarities, but also many differences in the protein sequences. Each subfamily of GRAS might have similar or related biological functions (Tian et al., 2004). For example, in model plants (rice and Arabidopsis), the GRAS family is clustered into 8 subfamilies: LISCL, PAT1, SCL3, DELLA, SCR, SHR, LS, and HAM (Tian et al., 2004). A total of 48 *SmGRAS* genes were divided into 13 subfamilies and distributed on 11 chromosomes unevenly in eggplant (Yang et al., 2022). DoGRAS proteins in *Dendrobium catenatum* could be classified into 10 groups together with *GRAS* proteins in Arabidopsis and rice, including DELLA, AtSCL3, AtSCL4/7, AtLAS, AtSCR, HAM, AtPAT1, AtSHR, LISCL and a new subfamily (unknown group) (Zeng et al., 2019). In *Camellia sinensis*, 52 CsGRAS proteins were classified into 13 groups upon the analysis of 33 proteins from Arabidopsis and 50 proteins from rice, including HAM, DELLA, AtSCL3, DLT, AtSCR, AtLAS, AtSCL4/7, AtSHR, AtPAT1, Os4, Os19, Os43, and LISCL (Wang et al., 2018). Based on 397 GRAS proteins from 8 plant species, a phylogenetic tree showed that these GRAS proteins were classified into 17 subfamilies, including PAT, RAD1, SCLA, SCR, DELLA, RAM1, SCL3, DLT, SCLB, LISCL, SCL4/7, LS, NSP2, HAM, NSP1, SCL32, and SHR (Cenci and Rouard, 2017). These studies indicate that the GRAS TF family is substantially diversified in different plant species. Moreover, due to differences in conserved domains, each subfamily has unique functions in plant growth and development, but the proteins of the same subfamily have similar functions. The members of the SCR and SHR subfamilies are primarily involved in regulating the growth of roots and leaves; for example, *AtSHR* and *AtSCR* could regulate the radial growth of roots and buds through the SCR/SHR complex (Sabatini et al., 2003). Furthermore, the *SCL3* subfamily gene maintains the functional pathway of gibberellin (GA) by weakening the DELLA inhibitory factor in the root cortex (Heo et al., 2011). Overexpressing transgenic plants of *PbGRAS89* and *PbGRAS99* from HAM subgroup increased callus formation from leaf explants compared to control Arabidopsis (Wang et al., 2022).

Recently, research has not only focused on the mining of the *GRAS* family in different plants, but also on the exploration of the functions of genes. Plant GRAS proteins are involved in various biological processes, such as root development (Benfey et al., 1993), shoot meristem maintenance (Wysocka-Diller et al., 2000), axillary meristem initiation (Schumacher et al., 1999), GA signal transduction (Peng et al., 1997; Silverstone et al., 1998), phytochrome A signal transduction (Bolle et al., 2000), and biotic/abiotic stress responses (Grimplet et al., 2016; Zhang et al., 2020; Wang et al., 2021; Liu et al., 2021a; Jaiswal et al., 2022). Moreover, recent studies have reported on the participation of GRAS proteins in the abiotic stress response in many plant species. For instance, the overexpression of GRAS protein SCL7 in *Populus euphratica* improved salt and drought tolerance in transgenic Arabidopsis plants (Ma et al., 2010). Expression of *RcGRAS* genes were induced by exogenous gibberellin (GA) and drought stress and played prevalent roles in regulations of plant growth and development, GA and drought stress signaling (Kumari et al., 2022). Overexpression of GRAS protein VaPAT1 from *Vitis amurensis* enhanced the salt, drought, and cold tolerance in transgenic Arabidopsis *via* the regulation of the expression of several stress-related genes (Yuan et al., 2016). Overexpression of *SlGRAS40* in tomato plants improved the tolerance to salt and drought stress *via* the enhancement of the ability to scavenge ROS (Liu et al., 2017). HcSCL13, a *Halostachys caspica* GRAS TF, could modulate salt stress tolerance in transgenic Arabidopsis through the regulation of plant growth and the activation of gene expression (Zhang et al., 2020). *GmGRAS37* was significantly upregulated under drought and salt stress conditions and abscisic acid treatment, and overexpression of *GmGRAS37* improved the resistance to drought and salt stress in soybean (Wang et al., 2020). *OsGRAS23* could positively regulate drought tolerance in transgenic rice *via* the modulation of amounts of stress-related genes (Xu et al., 2015), and *OsGRAS39* was highly induced under conditions of ABA or salt treatment (Dutta et al., 2021).

Birch is a species of deciduous hardwood that is widely distributed in the mid-high mountains of warm, temperate regions in the world. This widely grown tree is tolerant to drought, flooding, and light, and adapts well to many kinds of soil (Kang et al., 2014; Wang et al., 2014). GRAS is a TF family that is unique to higher plants, and it plays an important role in the growth and development of plants, especially in root formation, fruit development, plant response to adversity, and hormone signaling (Di Laurenzio et al., 1996; Silverstone et al., 1998; Tian et al., 2004; Chang et al., 2021). Although some *GRAS* genes (*GRASs*) have been characterized in many plant species and play many significant roles in response to abiotic stress, their genome-wide analysis and functional identification are still not completely studied and need to be resolved urgently in birch. At same time, there have been few reports on GRAS proteins involved in the abiotic stress response in *Betula platyphylla* (*B. platyphylla*). In this study, we performed a genome-wide analysis of the *GRAS* gene family in the birch plant and identified the characteristics of 40 *GRAS* genes. The expression patterns of *BpGRASs* in different tissues under salt stress were studied by reverse transcription quantitative polymerase chain reaction (RT-qPCR), and 26 *BpGRASs* were chosen for further analysis. The transient expression of 6 *BpGRASs* in birch was achieved using the transient transformation technique for studying the salt tolerance ability of *BpGRASs*. *BpGRAS34* gene was stably transformed and performed phenotype analysis and physiological indexes to further illustrating the tolerance to salt stress. This study will lay the foundation for clarifying the molecular mechanism of GRAS TFs in response to salt stress in birch plants and provide high-quality resistant genes for genetic engineering breeding of birch improvement.

## Materials and methods

### Plant materials and cultivation conditions

The seeds of birch were obtained from the State Key Laboratory of Tree Genetics and Breeding (Northeast Forestry University) and planted in a mixture of vermiculite and soil (v: v= 1:3) in pots. The birch seedlings were cultivated in a thermostatic greenhouse at a temperature of 25 ± 2°C, relative humidity of 65-70%, light intensity of 400 μmol·m^-2^s^-1^, and a light/dark photoperiod of 16 h/8 h.

### Bioinformatics analysis and chromosomal mapping

The DNA and protein sequences of 40 *BpGRASs* were searched from the birch genome database (GenBank accession: PRJNA285437). The ExPASy tool (http://www.expasy.org/tools/protparam.html) was used to predict the physicochemical parameters of the putative 40 BpGRAS proteins, such as the molecular weight (MW) and isoelectric point (pI). Based on the birch genome database, the chromosomal locations and duplications of 40 *BpGRASs* were physically mapped on the 14 chromosomes of birch.

### Identification of *GRAS* genes in birch plants and putative promoter *cis*-element analysis

The GRAS proteins of the other 5 plant species were obtained as followed ways: Arabidopsis from TAIR (http://www.arabidopsis.org/), rice from and PlantTFDB v5.0 (http://planttfdb.cbi.pku.edu.cn/), *Camellia sinensis* (tea) from Wang et al. (2018), *Phoenix dactylifera* (*P. dactylifera*) and *Theobroma cacao* (*T. cacao*) from Cenci and Rouard (2017). Phylogenetic analysis was performed with 40 BpGRAS proteins, 32 GRAS proteins from Arabidopsis, 38 from rice, 52 from tea plant, 59 from *P. dactylifera* and 44 from *T. cacao* using the neighbor-joining (NJ) method in the MEGA X program (Kumar et al., 2018; Zhang et al., 2021). Multiple sequence alignments of the selected 6 GRAS proteins of birch plants and 3 GRAS proteins of different species were performed using ClustalW (Thompson et al., 1997). The MEME/MAST program (http://meme-suite.org) was used for conserved protein motif analysis with a maximum of 20 motifs. Putative promoter sequences of 6 selected *BpGRASs* were obtained using 2 kb of a genomic sequence upstream of the translation start site of the 6 *BpGRASs* and were extracted from the birch genome database, respectively. *Cis*-acting elements were analyzed using the website PlantCARE (http://bioinformatics.psb.ugent.be/webtools/plantcare/html).

### Expression analysis of *BpGRASs* in different tissues under salt stress conditions

Two-month-old birch seedlings grown in the soil were watered with a solution of 200 mM NaCl for 3, 6, 12, 24, and 48 h, respectively, and treatment for 0 h was provided as the control. Three seedlings were collected after each treatment process. Total RNA of the root, stem, and leaf tissues of birch was extracted using the Universal Plant Total RNA Extraction Kit (BioTeke, Beijing, China). The extracted RNA was reverse-transcribed into cDNA with oligo (dT) primers in a reaction volume of 10 μL using a PrimeScript RT Reagent Kit (TaKaRa, Beijing, China) as a template for RT-qPCR. *Actin* (GenBank accession: MK388227) and *β-tubulin* (GenBank accession: MK388229) were used as reference genes for RT-qPCR analysis (Li et al., 2019). Each 20-μL volume of the reaction mixture included 10 μL of SYBR Green Real-time PCR Master Mix (Toyobo, Osaka, Japan), 2 μL of cDNA template (100 ng), and 0.5 μL of specific primers (10 μM). Amplification was performed by the reaction mixture at 94°C for 30 s, followed by 45 cycles at 94°C for12 s, 58°C for 30 s, 72°C for 45 s, and 82°C for 1 s during plate reading. Real-Time PCR Thermal Cycler-qTOWER³ (Analytik Jena AG, Jena, Germany) was used to perform RT-qPCR. Three replicates were used for each sample and the purity of the PCR products was evaluated using a melting curve. The expression levels were calculated from the cycle threshold using the 2^−ΔΔCt^ method (Livak and Schmittgen, 2001), and used to generate a heat map using R studio. The primers used are shown in **Supplementary Table 1**.

### Cloning and plasmid construction of *BpGRASs*

Total RNA of birch plants was extracted using the Universal Plant Total RNA Extraction Kit (BioTeke, Beijing, China). Total RNA was reverse transcribed into cDNA using a PrimeScript RT Reagent Kit (TaKaRa, Beijing, China), which was used as a template for PCR. We designed primers for the cloning of 6 *GRAS* genes from different tissues of birch plants. All the primers are listed in **Supplementary Table 2**. The PCR procedure was as follows: the reaction mixture at 94°C for 3 min, subjected to 30 cycles at 94°C for 30 s, 58°C for 30 s, 72°C for 1 min and 30 s, and 72°C for 7 min. The PCR products were purified and recovered using a Cycle Pure Kit (Omega, Norcross, GA, America). The obtained full-length cDNA of *GRAS* genes was inserted into the pROKII plasmid, regulated by the CaMV 35S promoter (35S:BpGRAS), and inverted-repeat cDNA sequences of *GRAS* genes were constructed into the pFGC5941 RNAi vector (pFGC : BpGRAS) for silencing gene expression. The recombinant plasmids exhibiting overexpression (35S:BpGRAS) and inhibited expression (pFGC : BpGRAS) of *BpGRASs* were transformed into *Agrobacterium tumefaciens* strain EHA105 *via* electroporation.

### Plant transformation for analysis of expression and physiological determinations under salt stress treatment

The recombinant plasmids exhibiting an overexpression (35S:BpGRAS) and inhibited expression (pFGC : BpGRAS) of *BpGRASs* were transferred into 4-week-old birch seedlings *via* high-efficiency transient transformation by the method of Ji et al. (2014), using the empty vector (pROKII) as a control. Stable transgenic birch lines were obtained using method of *Agrobacterium tumefaciens*-mediated transformation (Guo et al., 2017) with recombinant plasmids exhibiting an overexpression of *BpGRAS34* with the wild-type birch (WT) as the control. Whole transient-transformation plants of overexpression (OE), inhibited-expression (IE) and control plants were treated with 1/2 MS or 1/2 MS containing 150 mM NaCl for 24 h for RT-qPCR and the measurement of physiological indexes. The RNA of whole birch plants of stable transgenic lines was extracted and reverse transcribed into cDNA for RT-qPCR to analyze expression levels, respectively. The primers used were listed in the **Supplementary Table 2**. Stable transgenic lines were treated with 1/2 MS or 1/2 MS containing 150 mM NaCl for 24 h to measure physiological indexes. The electrolyte leakage assay was performed and the malondialdehyde (MDA) content was measured in accordance with the method described by Ji et al. (2014) and Wang et al. (2010). The level of ROS was determined using the Plant ROS Elisa Kit (SenBeiJia, Nanjing, China) and hydrogen peroxide (H_2_O_2_) content was measured with the Hydrogen Peroxide assay kit (Nanjing Jiancheng, Nanjing, China). Superoxide dismutase (SOD) and peroxidase (POD) activities were detected using the protocols described by Asada et al. (1973) and Wang et al. (2010), and proline content was measured using the method described by Bates et al. (1973). Three biological replicates were performed in each experiment.

### Biological staining and phenotype analysis

After treatment with 1/2 MS containing 150 mM NaCl for 2 h, the leaves of birch seedlings were used for biological staining. Cell death was observed *via* Evans blue staining, using the protocol described by Kim et al. (2003). The H_2_O_2_ and superoxide ${\text{O}}_{2}^{-\cdot }$ contents were determined *via* diaminobenzidine (DAB) and nitroblue tetrazolium (NBT) staining of the detached leaves based on the methods described by Zhang et al. (2011). After grown in pots with the soil for two months, stable transgenic birch plants and wild-type birch plants were used for phenotype analysis watered with 200 mM NaCl for 10 days. Plants treated with water were served as the control.

### Statistical analyses

Statistical analyses were performed using SPSS (IBM SPSS 22, IBM Corp., Armonk, NY, USA). Data were analyzed using the Student’s t-test. The differences were significant if p< 0.05; this is represented by the * symbol in figures. Three biological replicates were generated for statistical analyses.

## Results

### Identification and chromosomal mapping of GRAS TFs in birch plants

A total of 40 *BpGRASs* were obtained from the birch genome database and identified. Their physicochemical properties were further analyzed using ExPasy (**Supplementary Table 3**). Most of these proteins had typical GRAS domains containing approximately 350 amino acids (aa), while the GRAS domains of BpGRAS6 and BpGRAS8 were severely truncated and had less than 150 aa. The predicted lengths of the 40 BpGRAS proteins and their MWs (kDa) ranged from 182 aa to 830 aa and 21.07 kDa to 90.86 kDa, respectively. For most BpGRAS proteins, the theoretical pI values ranged from 4.89 to 6.88; four of the BpGRAS proteins were alkalescent, indicating that most BpGRAS proteins were acidulous and may cause variations in BpGRAS protein functions in different environments. The grand average of hydropathy (GRAVY) of all BpGRAS proteins (ranging from -9.773 to -0.102) suggested that all BpGRAS proteins are hydrophilic; these results were similar to the results obtained for GRAS proteins in *Prunus mume* (Lu et al., 2015). Most predicted instability index values exceeded 40 (ranging from 40.65 to 61.17), indicating that a majority of BpGRAS proteins were unstable, except for BpGRAS35 (34.86) and BpGRAS36 (39.39). Most *BpGRASs* had no introns, which indicates that the sequences of *BpGRASs* are conservative at a certain extent.

**Figure 1:**
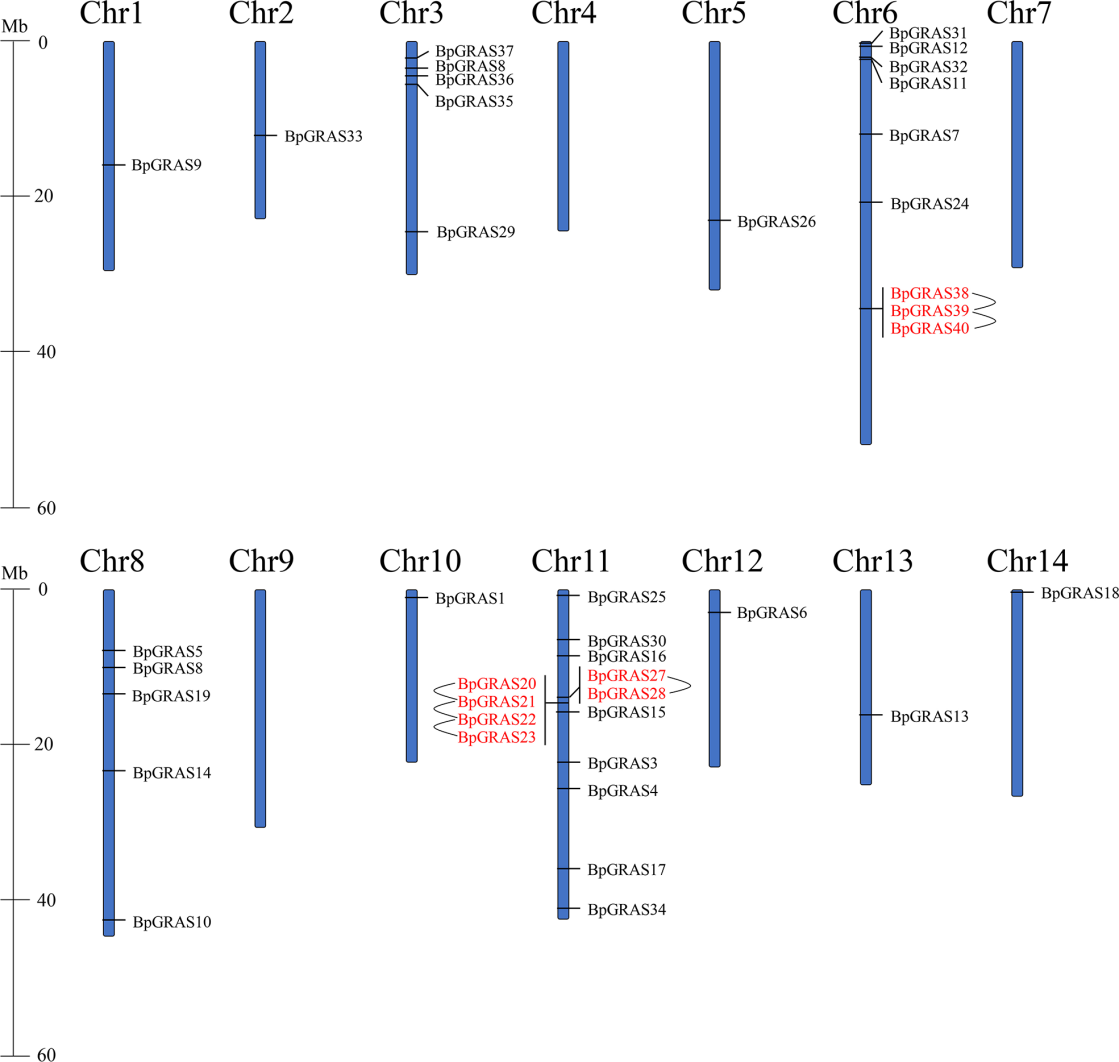
Positions and distributions of *BpGRASs* family members on 14 chromosomes of birch. Vertical bars represent the chromosomes of birch and the chromosome number is indicated beside each chromosome. Tandem duplicated genes are emphasized with red color and connected with black lines. The scale on the left represents chromosome length.

The identified 40 *BpGRASs* were further mapped and positioned on 14 birch chromosomes (Chr1 to Chr14) (**Figure 1**). In general, 40 *BpGRASs* had uneven distributions on 14 birch chromosomes expect *BpGRAS2*. The densities of *BpGRASs* distributed on birch chromosomes were different and uneven among different chromosomal regions. There were no *BpGRASs* found on the Chr4, 7 and 9. Chr11 contained the most *BpGRASs* and 14 *BpGRASs* (35%) were distributed on this chromosome, followed by Chr6 (9, 22.5%) and then both Chr3 and Chr8 (5 each, 12.5%). Only 1 *BpGRASs* (2.5%) was located on the Chr1, 2, 5, 10, 12, 13 and 14. We speculated that there was no obvious connection and correlation between *GRASs*’ number and chromosome length according to the previous research (Chen et al., 2019; Liu et al., 2019). A tandem duplication event of genes was defined that a chromosomal region within 200 kb contained 2 or more genes, and plays a vital role for gene family in occurrence further expansion of novel functions (Fan et al., 2021b). Six tandem duplication events were found on the Ch6 and 11 including *BpGRAS20*/*BpGRAS21*, *BpGRAS21*/*BpGRAS22*, *BpGRAS22*/*BpGRAS23*, *BpGRAS27*/*BpGRAS28*, *BpGRAS38*/*BpGRAS39, BpGRAS39/BpGRAS40*, involving total 9 *BpGRASs*. All the genes involved in tandem duplication events belonged to the same subfamily. Except *BpGRAS38*, *BpGRAS39* and *BpGRAS40* from PAT subfamily, 6 genes in tandem duplication events belonged to LISCL subfamily, indicating that LISCL group played an important role in expansion of *GRASs* as the largest subfamily (Fan et al., 2021a).

### Phylogenetic analysis

**Figure 2:**
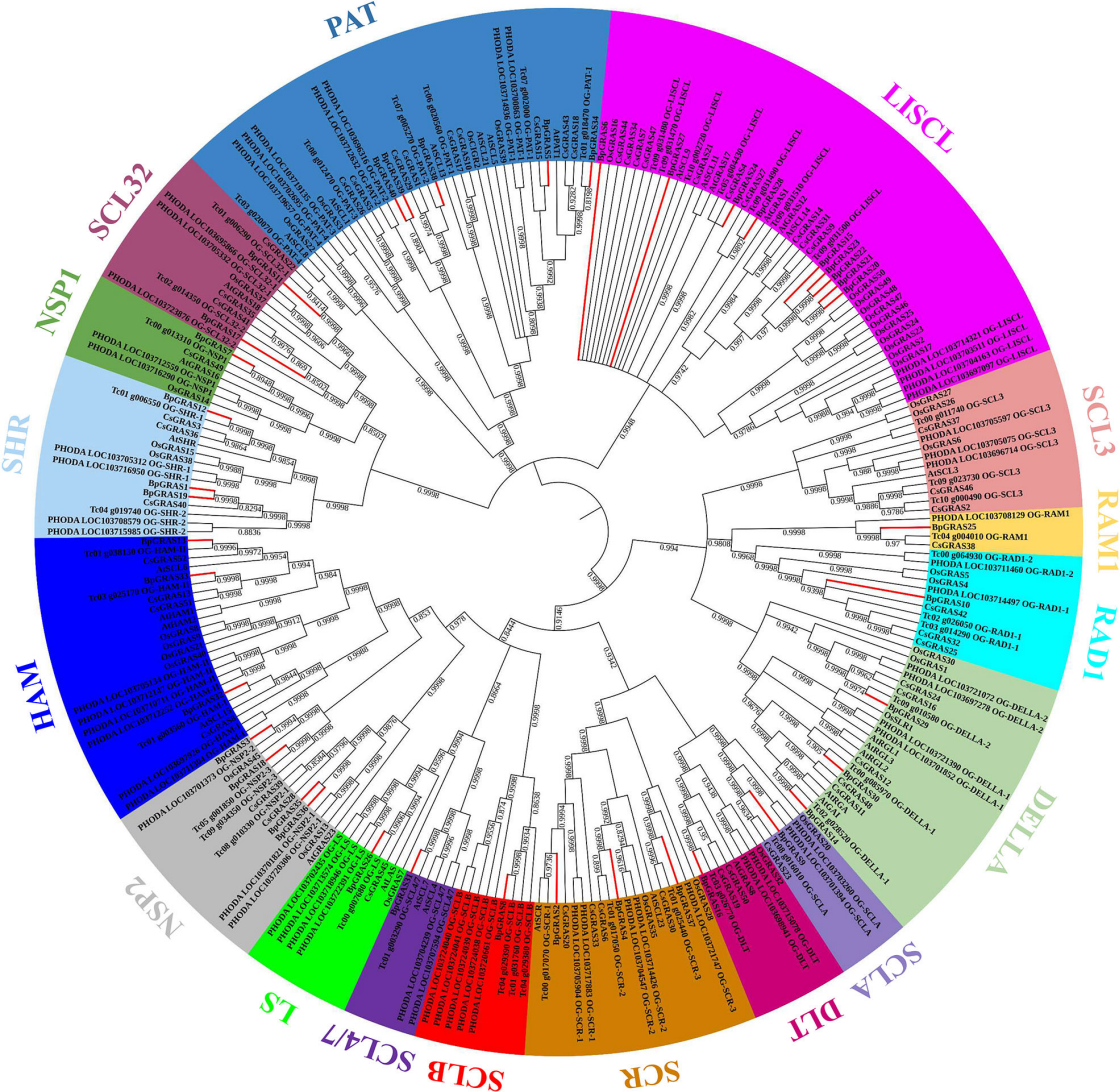
Phylogenetic analysis of the BpGRAS proteins and GRAS proteins obtained from Arabidopsis, rice, tea plant, *P. dactylifera* and *T. cacao*. Total 265 GRAS proteins obtained from 6 plant species were aligned. The unrooted NJ tree was constructed using MEGA X. All the BpGRAS proteins were emphasized with red branches.

Based on the latest genome assemblies, we found 265 putative *GRAS* genes: 40 in birch, 32 in Arabidopsis, 38 in rice, 52 in tea plant, 59 in *P. dactylifera* and 44 in *T. cacao*, respectively. An unrooted phylogenetic tree was constructed using MEGA X using the NJ method with a bootstrap value of 100 for the identification of the evolutionary relationships among the 40 BpGRAS proteins, 32 GRAS proteins of Arabidopsis, 38 of rice, 52 of tea plant, 59 of *P. dactylifera* and 44 of *T. cacao* (**Figure 2**). Phylogenetic analysis showed that these 265 GRAS proteins could be divided into 17 groups (LISCL, SCL3, RAM1, RAD1, DELLA, SCLA, SCLB, DLT, SCR, SCL4/7, LS, NSP1, NSP2, HAM, SHR, SCL32 and PAT). These findings revealed the basic role of GRAS family proteins in the evolution and development of different plant species and were similar to those of previous reports of some other plant species, including *Vitis vinifera*, *Musa acuminata*, *Coffea canephora* and so on (Cenci and Rouard, 2017). BpGRAS proteins were distributed in 17 subfamilies unevenly, and most of them belonged to LISCL subfamily (9 members). Only 1 BpGRAS protein could be observed in subfamilies RAM1, RAD1, SCLA, DLT, SCLB, SCL4/7, LS and NSP1, respectively. However, no BpGRAS protein belonged to SCL3 group. The phylogenetic tree showed that some BpGRAS proteins were closely related to those of other species (bootstrap support ≥ 80), indicating that these BpGRAS proteins might be orthologous to the GRAS proteins of other plants and have similar functions.

### Motif analysis of *BpGRASs*

**Figure 3:**
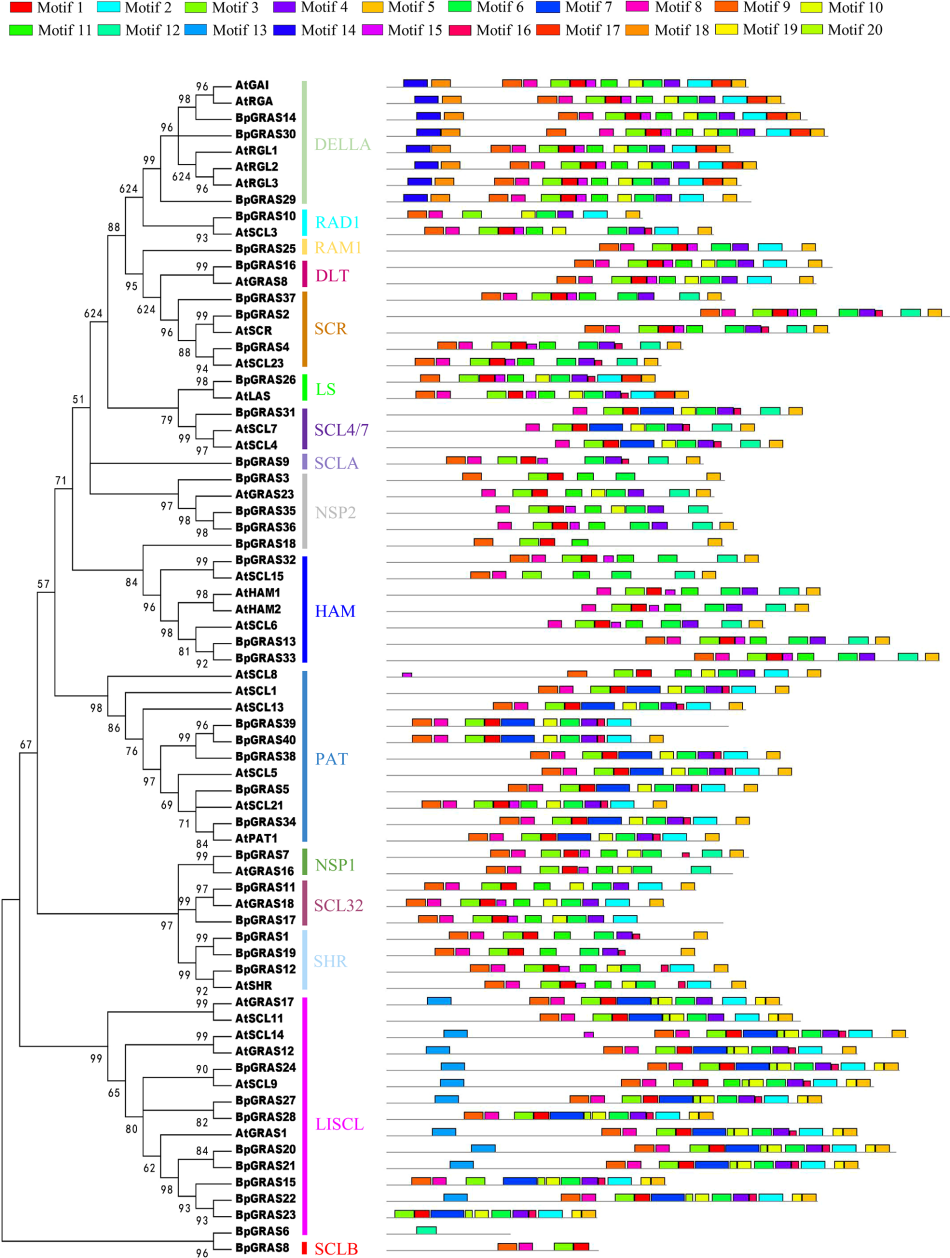
Putative motifs in each GRAS protein from birch and Arabidopsis. Schematic representation of the conserved motifs elucidated by MEME. Each motif is represented by a number in the colored box. The black lines represent non-conserved sequences.

To further explore the sequence features of GRAS TFs in birch plants, we performed a comparative analysis of the conserved motifs between birch and Arabidopsis (**Figure 3**). The structural details of the GRAS proteins were analyzed *via* 20 motifs predicted by the MEME program. In general, similar motif compositions could occur among GRAS proteins of the same subfamily, suggesting that GRAS proteins in the same subfamily may have similar functions. Almost all GRAS proteins contained motifs 1, 3, 5, 6 and 8, indicating that these motifs were highly conserved and may play important roles in the GRAS family. Motifs 14 and 18 were only distributed in DELLA subfamily; motifs 13, 19 and 20 were only found in LISCL subfamily; motif 7 was only distributed in SCL4/7, LISCL and PAT subfamilies; motif 16 was absent in DELLA, RAM1, DLT, NSP2, HAM, SCL32, and SCLB subfamilies. Motif 3 was found in all proteins except BpGRAS6; BpGRAS6 contained only motif 12, and BpGRAS8 contained only motifs 1, 3, 8 and 9. Some motifs were distributed only at certain locations in the pattern. For example, motif 5 was always distributed at the end of the pattern, and motif 9 was almost always distributed at the start. The functions of most of these conserved motifs still need to be understood. From the differences in the distribution of these motifs between subfamilies, it can be seen that GRAS proteins of different subfamilies may have different functions; meanwhile, different genes from the same subfamily also exhibited a different distribution of motifs, indicating that the functions of such genes may also be different. In specific GRAS protein subfamilies, similar motifs tended to be clustered together, indicating that there might be functional similarities among those proteins.

### Expression patterns of *BpGRASs* in different tissues under salt stress conditions

**Figure 4:**
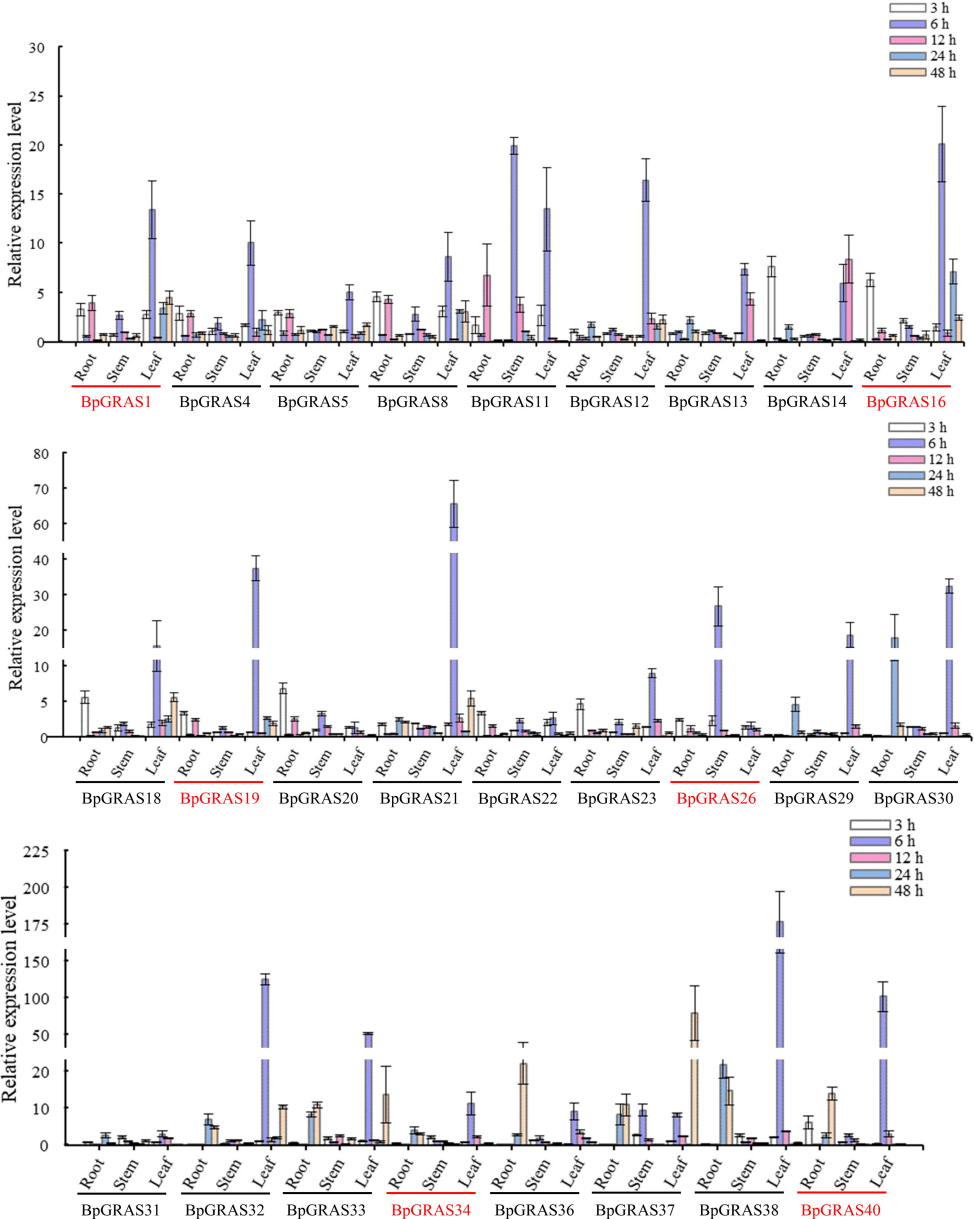
Expression analysis of selected *BpGRASs* using RT-qPCR. The expression patterns of *BpGRASs* in the roots, stems, or leaves of birch plants in response to treatment with NaCl (200 mM). The expression of *BpGRASs* under normal conditions (0 h) was designated as 1, in order to standardize the expression level of *BpGRASs* under salt stress conditions. Three independent experiments were performed, and data are means ± SD from the three experiments. Six *BpGRASs* selected for further analysis were emphasized with red colors.

**Figure 5:**
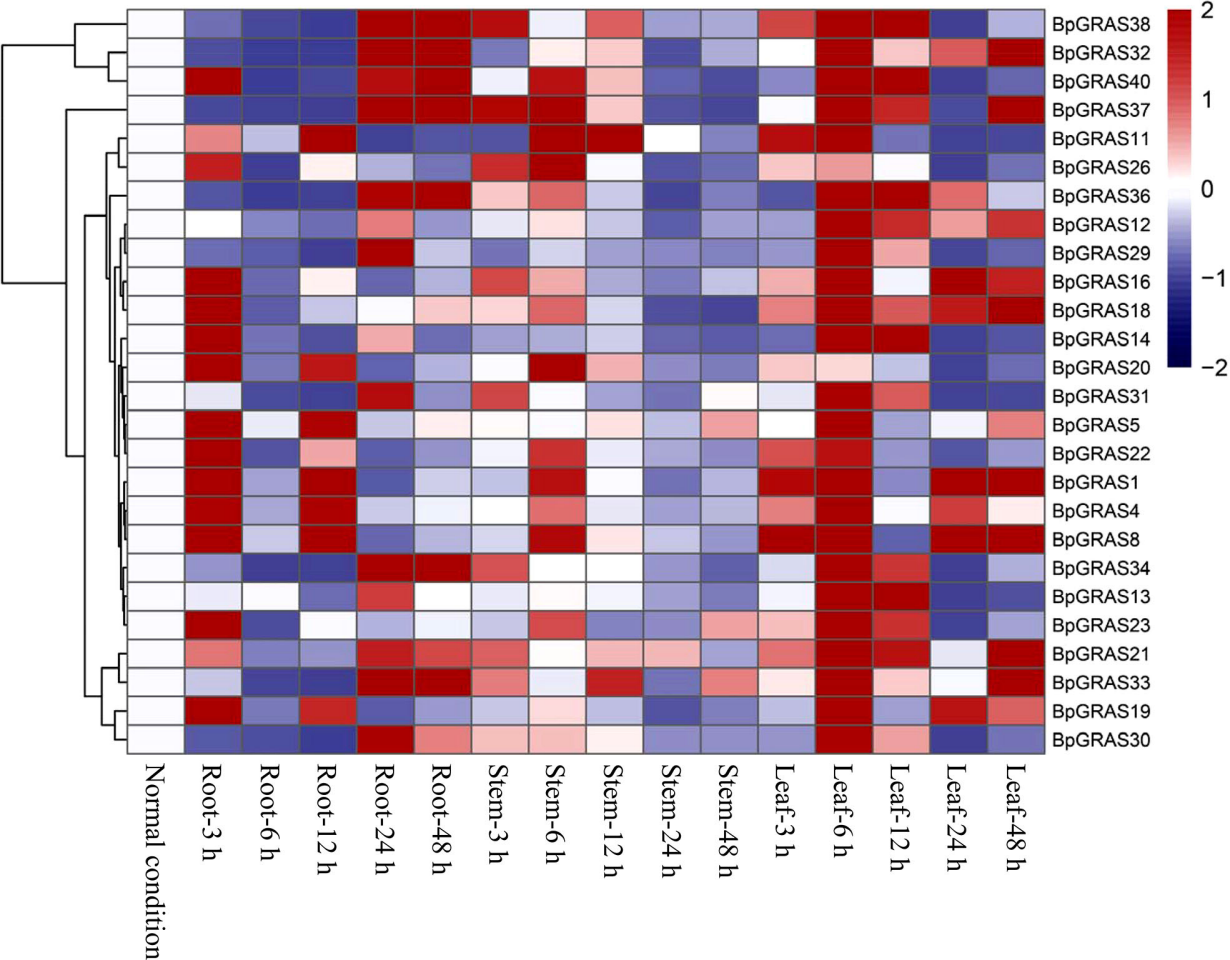
Heat map of the expression profiles of 26 *BpGRASs*. Heat map of the expression profiles of all *BpGRASs* in different birch tissues at different time points under 200 mM NaCl stress treatment. The color scale represents the log2-transformed gene relative expression compared to that observed under normal conditions (0 h): blue to red colors denote the low to high level of relative expression.

Based on the latest birch genome assembly results, 26 *BpGRASs* were successfully cloned for further gene function studies. Thus, different tissues (from the root, stem, and leaf) were collected after treatment with 200 mM NaCl for 3, 6, 12, 24, or 48 h for RT-qPCR, to analyze the expression patterns of the 26 *BpGRASs* (**Figures 4** and **5**). The results showed that all genes were expressed in the root, stem, and leaf tissues at each time point, which indicated that the *GRAS* genes might play a role in plant growth and development. Most of the *BpGRASs* (17 genes) were highly induced by salt stress at 6 h and were significantly expressed in the leaf tissues except *BpGRAS26* in the stem. *BpGRAS13* was significantly induced in the leaf at 6 and 12 h after salt treatment (**Figures 4** and **5**). Only the expression level of *BpGRAS37* peaked under salt stress conditions at 48 h in the leaf (**Figures 4** and **5**). In the root tissue, *BpGRAS20* and *BpGRAS36* were expressed significantly at 3 and 48 h, respectively. On the other hand, *BpGRAS11* was also significantly expressed at 6 h in the leaf and stem tissues, and *BpGRAS30* was highly induced by salt stress at 6 h in the leaf and 24 h in the root. These results indicated that high levels of most of the *BpGRASs* were induced in the leaves in response to salt stress at 6 h.

### Multiple sequence alignment and *cis*-acting element analysis of *BpGRASs*

Six *BpGRASs*, i.e., *BpGRAS1*, *BpGRAS16*, *BpGRAS19* (GenBank accessions: MN117546-MN117548), *BpGRAS26*, *BpGRAS34*, and *BpGRAS40* (GenBank accessions: MZ062900-MZ062902), were selected for further study because they were significantly upregulated under salt treatment conditions and had better expression patterns as shown in **Figure 3**. These 6 BpGRAS proteins, which exhibited a high level of homology to GRAS proteins of Arabidopsis and rice, were selected for multiple sequence alignment analysis. The results indicated that the GRAS proteins of birch and other plant species shared a highly conserved binding domain at the C-terminus (**Supplementary Figure 1**), and the six selected BpGRAS proteins contained certain GRAS domains that are characteristically found in the GRAS family (Pysh et al., 1999).

**Figure 6:**
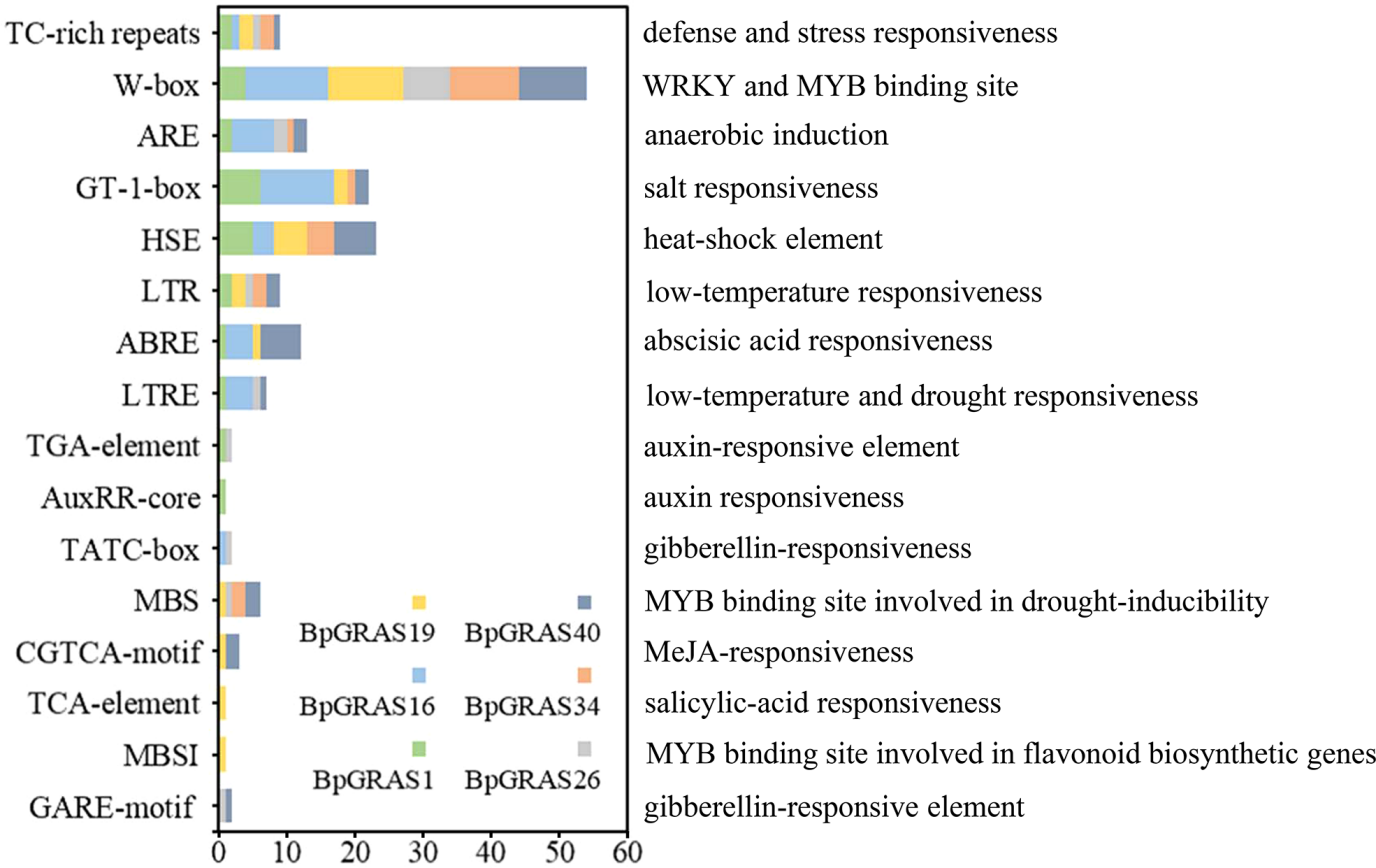
The cis-acting elements of 6 GRAS gene promoters in birch related to environmental stress and phytohormone signals. The X-axis indicates the number of each of the cis-acting elements; the Y-axis indicates the different cis-acting elements.

The distribution of *cis*-acting elements in promoters may be responsible for the diversity of functions and expression patterns of different genes. *Cis*-acting elements were identified from the 2-kb region upstream of the start codon in the promoters of 6 selected *GRAS* genes (*BpGRAS1*, *BpGRAS16*, *BpGRAS19*, *BpGRAS26*, *BpGRAS34* and *BpGRAS40*), for further identifying their role in the development of tolerance to salt-shock-induced stress (**Figure 6**).

Six *cis*-acting elements were analyzed and found to be involved in response to abiotic stress or phytohormone conduction; these included 6 stress-response elements and 10 phytohormone-related elements. All 6 genes contained 7 to 11 *cis*-acting elements as shown in **Figure 6**. Each of these 6 *GRAS* genes had at least one element related to the stress response, such as TC-rich repeats, GT-1-box, HSE, LTR, and LTRE, and played a role in generating a stress response. MBS had a drought inducibility-related function because it acted as the binding site of MYB. Meanwhile, 10 phytohormone-related elements of the 6 *GRAS* genes occurred in most plant hormones; these included the elements that played a role in the abscisic acid-responsiveness (ABRE), auxin-responsiveness (TGA-element, AuxRR-core), gibberellin-responsiveness (TATC-box, GARE-motif), jasmonic acid-responsiveness (CGTCA-motif), and salicylic acid-responsiveness (TCA-element). These results suggested that the 6 *BpGRASs* might confer tolerance to abiotic stresses such as salt, cold, and drought, and participate in the plant growth and development process.

### Overexpression of *6 BpGRASs* can decrease cell death

Six *BpGRASs* were used for constructing *BpGRAS* overexpressing and inhibiting recombinant vectors, and transiently transformed birch plants were collected for RT-qPCR *via* high-efficiency transient transformation (Ji et al., 2014). The results showed that birch plants exhibiting transient overexpression or the knockdown of the 6 *BpGRASs* had been obtained successfully, with highest expression levels of all the OE plants among variety of transiently transformed plants induced by salt treatment (**Supplementary Figure 2**).

**Figure 7:**
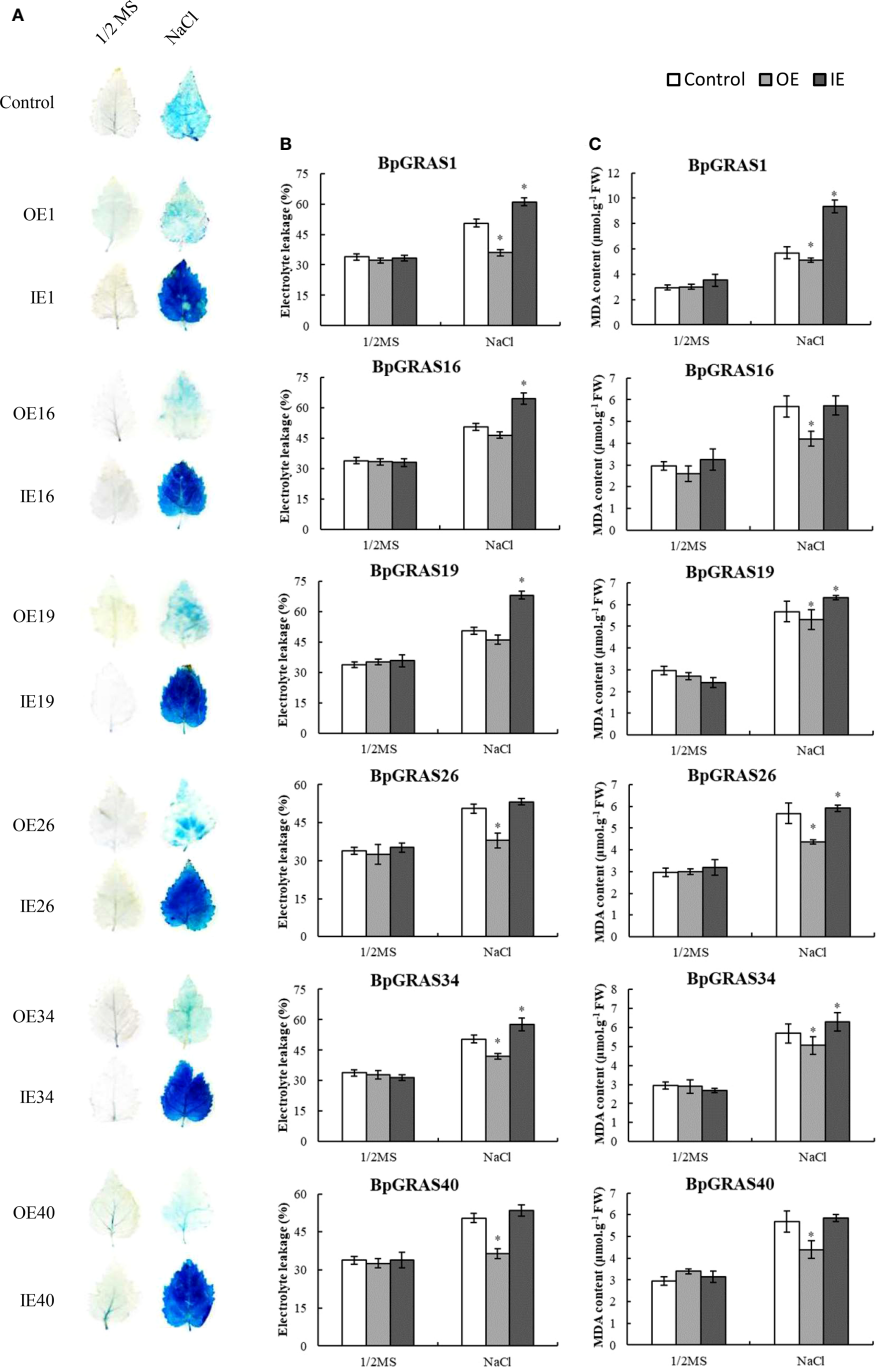
Detection of cell death, electrolyte leakages, and MDA contents in OE, IE, and control plants. **(A)** Birch plants treated with 150 mM NaCl and stained with Evens blue to visualize cell death. **(B)** Comparison of electrolyte leakage rates. **(C)** MDA contents. Data represents means ± SD values from three independent experiments. * significant (P< 0.05) difference was observed, compared to control plants. Control: birch plants transformed with empty pROKII; OE: birch plants exhibiting overexpression of *BpGRAS*; IE: birch plants exhibiting inhibited expression of *BpGRAS*.

Physiological determinations of 6 *BpGRASs* were performed for further identifying whether 6 *BpGRASs* conferred tolerance to salt stress. Cell death is often measured to detect stress tolerance in plants. Evans blue staining was performed to study cell death after salt stress treatment (Zhang et al., 2011). Under normal growth conditions, three types of plants, i.e., plants exhibiting the overexpression and inhibition of 6 transiently transformed *BpGRASs*, and control plants (Control) showed a consistent level of staining. Under salt stress, OE plants were stained more lightly than control and IE plants, and the staining intensity of IE plants was the highest (**Figure 7A**). Under salt stress, the electrolyte leakages of IE plants of these 6 *BpGRASs* were higher than that of control plants, while the OE plants had the lowest electrolyte leakages (**Figure 7B**). An assessment of the MDA contents showed that there were no significant differences in OE, IE and control plants of the three transient transgenic plants under normal growth conditions. However, the MDA content of OE plants was the lowest, compared to the control plants after salt stress treatment (**Figure 7C**). These results showed that overexpression of *BpGRASs* resulted in minimal levels of cell death, indicating that overexpression of *BpGRASs* resulted in better salt stress tolerance in birch plants.

### Overexpression of *BpGRAS34* improves transgenic birch salt tolerance

**Figure 8:**
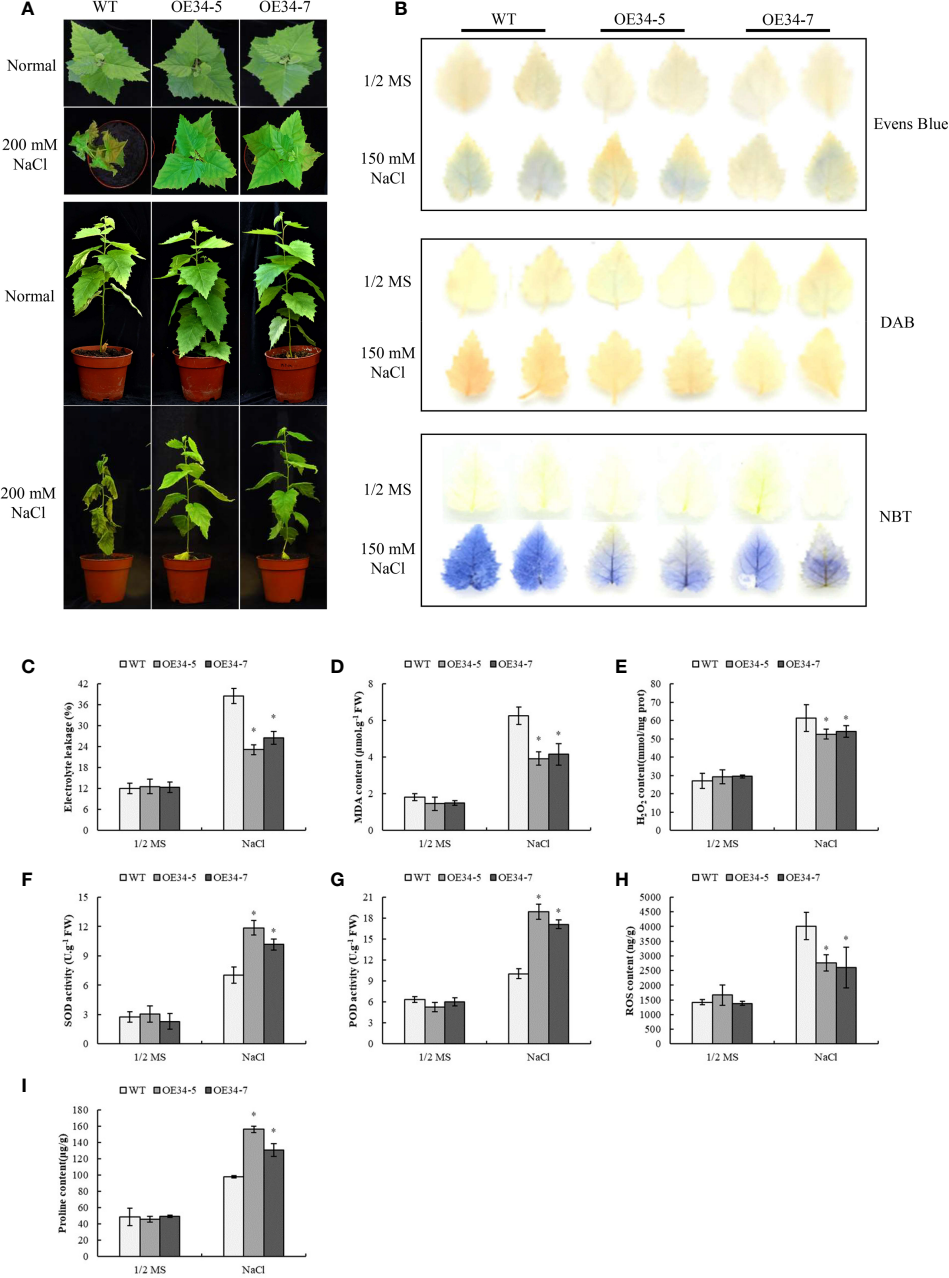
Phenotype analysis, biological staining and physiological determinations of two stable transgenic lines of *BpGRAS34* and WT plants. **(A)** Phenotype of *OE34-5*, *OE34-7* and WT plants treated with 200 mM NaCl. **(B)** *OE34-5*, *OE34-7* and WT plants treated with 150 mM NaCl and stained with Evens blue, DAB and NBT to visualize cell death, H_2_O_2_ and ${\text{O}}_{2}^{-\cdot }$ accumulation. **(C–I)** Electrolyte leakage rates **(C)**, MDA content **(D)**, H_2_O_2_ content **(E)**, SOD and POD activity **(F, G)**, ROS content **(H)** and proline content **(I)** of *OE34-5*, *OE34-7* and WT plants treated with 150 mM NaCl. WT: wild-type birch plants; *OE34-5* and *OE34-7*: two stable transgenic lines of *BpGRAS34* overexpression. * significant (P< 0.05) difference was observed compared with WT plants. Three independent experiments were performed in physiological determinations, and data are means ± SD from the three experiments.

To study whether overexpression of *BpGRASs* could improve the salt tolerance of birch, *BpGRAS34* was randomly selected to obtain stable transgenic overexpression plant. We obtained 11 transgenic lines of *BpGRAS34* overexpression and detected their expression levels *via* RT-qPCR. Lines *BpGRAS34-5* and *BpGRAS34-7* (*OE34-5* and *OE34-7*) were high expressed compared to the other lines (**Supplementary Figure 3**) and selected for next measurement. Phenotype analysis could intuitively show degree of injury of plants under stress conditions. Under the normal condition, there were not substantially different phenotypes of control plants and *OE34-5* and *OE34-7* plants, suggesting that *BpGRAS34* could not affect growth and phenotype of birch. However, leaves of control plant got wilting while *OE34-5* and *OE34-7* plants remained alive and greener after salt treatment (**Figure 8A**). Evens blue staining was used to investigate cell death under salt stress. Compared with the control plant, OE lines reduced staining after salt stress, indicating lower cell death (**Figure 8B**). As well as the results of electrolyte leakages and MDA contents, *OE34-5* and *OE34-7* plants were lower than control plants under salt stress treatment; while there was no significant difference between the control plants and overexpression plants (**Figures 8C, D**). These results suggested that *OE34* reduced cell death under salt treatment.

ROS plays an important role in the evaluation of plant stress tolerance (Gechev et al., 2006). NBT and DAB staining were used to determine the level of ROS accumulation *via* the detection of ${\text{O}}_{2}^{-\cdot }$ and H_2_O_2_ — the two main components of ROS. NBT and DAB staining, H_2_O_2_ content, SOD and POD activities and ROS content were evaluated to view if *OE34* can improve ROS scavenging. No obvious difference in NBT and DAB staining was observed among *OE34* and control lines under control conditions. However, compared with control plants under salt treatment condition, the results of histochemical staining of birch tissues using NBT and DAB showed that the levels of both ${\text{O}}_{2}^{-\cdot }$ and H_2_O_2_ in OE plants were lower than those in control plants (**Figure 8B**). The results of these analyses were consistent with results indicating H_2_O_2_ content and ROS accumulation levels. There was no obvious difference between OE and control lines in the measurement of H_2_O_2_ content. *OE34-5* and *OE34-7* had lower H_2_O_2_ content than control plants after salt treatment (**Figure 8E**), indicating that *OE34* reduced H_2_O_2_ accumulations in birch under salt stress condition. At the same time, transiently transformed plants of 6 *BpGRASs* also showed better ability of decreasing the H_2_O_2_ accumulations in the **Supplementary Figures 4A, B**. Furthermore, SOD and POD are two major ROS scavenging enzymes whose activities have extensively been used as an indicator of stress tolerance in plants (Zang et al., 2015). Under normal growth conditions, the activities of SOD and POD in *OE34* plants were not different from those in control plants. However, the activities of SOD and POD in *OE34* plants were significantly higher than those in the control plants under salt stress (**Figures 8F, G**). Additionally, *OE34-5* and *OE34-7* resulted in the lower ROS levels compared to those observed in the control plants (**Figure 8H**). Similarly, these analyses were repeated for transiently transformed plants of 6 *BpGRASs* and the results were consistent (**Supplementary Figure 4C** and **Supplementary Figure 5A, B**). These outcomes indicated that a decrease in ROS accumulation was attributable to the overexpression of *BpGRASs* in birch plants, thereby enhancing SOD and POD activity under salt stress.

In addition, we compared the proline levels in *OE34-5*, *OE34-7* and WT plants, to investigate whether *BpGRASs* can regulate proline biosynthesis under salt stress conditions (**Figure 8I**). Under normal growth conditions, the proline levels in *OE34-5*, *OE34-7* and control plants were almost the same; however, the proline content in *OE34* plants subjected to salt stress treatment was significantly higher than that of the control (**Figure 8I**). When exposed to salt condition, the similar results were found overexpression of 6 *BpGRASs* in transiently transformed plants, which could increase proline content (**Supplementary Figure 5C**). Thus, the overexpression of *BpGRASs* can increase the proline content in birch plants under salt stress conditions. The above-mentioned results preliminarily showed that overexpression of *BpGRAS34* can improve tolerance to salt by decreasing cell death, enhancing ROS scavenging ability and increasing proline content, further proved that overexpression of *BpGRASs* can enhance salt tolerance of birch, consistent with *cis*-acting analysis (**Figure 6**).

## Discussion

Plants use certain adaptive measures to deal with imminent pressure, mainly *via* the regulation of genes (Lin et al., 2017). GRAS family proteins are plant-specific TFs that play a crucial part in regulating the growth, development, and stress response (Pysh et al., 1999). Birch is a kind of deciduous hardwood tree species and it plays vital role in ecological and evolutionary importance. Therefore, it is important to understand the expression patterns of *GRAS* genes, which play a key role in signal transduction in birch plants. An analysis of their spatial and temporal regulation processes would help us identify candidate genes for the improvement of the abiotic tolerance of birch plants in the current environment. GRAS TF family members have been identified in multiple plants, such as Arabidopsis (Tian et al., 2004) and *Fagopyrum tataricum* (Liu et al., 2019). In this study, we performed a genome-wide analysis of the *GRAS* gene family in the birch plant and identified the characteristics of 40 GRAS proteins, most of which had typical GRAS domains containing approximately 350 aa (**Supplementary Table 3** and **Supplementary Figure 1**). The results were consistent with those observed in a previous study, in which most of the GRAS proteins shared a conserved C-terminal GRAS domain (Liu and Wang, 2021). The GRAS group was reported to originate in bacteria, and then expand into eukaryotic genomes *via* the possible retroposition of intronless genes by horizontal gene transfer and repeat generation (Huang et al., 2015). This is in accordance with the results of our study, which showed that 29 genes of a total of 40 *BpGRASs* were intronless (**Supplementary Table 3**). Moreover, the GRAVY index and a pI value of less than 7 in a majority of the GRAS proteins in **Table S3** indicated that the GRAS group might be involved in protein-protein interactions (Jing et al., 2017) that were very specific to GRAS proteins, because proteins with low pI values tend to minimize the chances of non-specific interactions with nucleic acids and other acidic proteins (Takakura et al., 2015). Chromosomal position showed that the identified 40 *BpGRASs* were distributed on the 14 chromosomes of birch unevenly except *BpGRAS2* (**Figure 1**). However, there were no *BpGRASs* found on the Chr4, 7 and 9. These results are similar to other studies, such as *SbGRASs* were not found on Chr7 in sorghum and 57 *PgGRASs* were located on 7 chromosomes of pearl millet except *PgGRASs*, which may be due to fragment loss or chromosomal shift and gene duplication events during the course of evolution (Fan et al., 2021b; Jha et al., 2021). Tandem duplications were considered to be one of representative main causes of gene family expansion in plants (Cannon et al., 2004; Zhu et al., 2014). Six tandem duplication events were found in this study involving total 9 *BpGRASs* and the genes involved in the same tandem duplication events belonged to the same subfamily (**Figure 1**). Phylogenetic analysis facilitated the clustering of 265 GRAS proteins into 17 subfamilies (**Figure 2**). Tandem repeats of closely related GRAS homologs were commonly observed during conserved motif analysis (**Figure 3**). Remarkably, motif compositions may be similar among GRAS proteins of the same subfamily, suggesting that GRAS proteins in the same subfamily may have similar functions. However, motifs within different subfamilies were varied, which might be attributable to the diverse biological functions of *GRASs*. As reported previously, GRAS proteins were randomly distributed in the phylogenetic tree (To et al., 2020; Wang et al., 2020), and had similar functions when they were in the same subfamily (Liu and Wang, 2021).

Previous studies have shown that GRAS proteins in different species had different spatial and temporal expression patterns. For example, the expression of four *MeGRAS* genes (*MeGRAS2, 11, 22*, and *32*) peaked at 6 h but decreased after 3 d in three *Cassava* varieties subjected to salt treatment (Shan et al., 2020). Besides, in orchardgrass, the expression levels of *DgGRAS5*, *DgGRAS28*, *DgGRAS31*, *DgGRAS42*, and *DgGRAS44* fluctuated at the seeding stage, compared to the stable expression pattern observed at the mature stage (Xu et al., 2020). Collectively, we identified the expression patterns of 26 *BpGRASs*, most of which were substantially induced by salt stress at 6 h and significantly expressed in the leaf tissues (**Figures 4**, **5**). This suggests that most *BpGRASs* were highly induced in the leaves in response to salt stress at 6 h and presented tissue-specific expression patterns (Khan et al., 2022). Similarly, *AtSHR*, which plays a key role during the visible and flowering stages of leaves in Arabidopsis (Wang et al., 2011) and is a homologous gene of *BpGRAS1* and *BpGRAS19*, was highly expressed in leaves.

To further confirm that *BpGRASs* can respond to salt stress, the analysis of *cis*-acting elements in the promoters of 6 *BpGRASs* was carried out. The results illustrated that many elements were involved in response to abiotic stresses, such as salt, cold, and drought. Among these *cis*-acting elements related to abiotic stress, TC-rich repeats and W-box had functions related to the stress response and was WRKY and MYB binding site, respectively; both these were observed in all 6 *BpGRASs* (**Figure 6**). Birch plants with transient overexpression or knockdown of 6 *BpGRASs* were obtained successfully using RT-qPCR and high-efficiency transient transformation (**Supplementary Figure 2**). Besides, the expression of *BpGRASs* were greatly induced by salt stress conditions in the birch plant (**Supplementary Figure 2**), indicating that *BpGRASs* may play a role in abiotic stress responses. Many studies have also analyzed *GRAS* expression patterns through RT-qPCR under abiotic stress conditions; for example, *MeGRAS* expression profiles were analyzed under different abiotic stresses (drought, salt, cold, and H_2_O_2_) (Shan et al., 2020); *GmGRAS* gene expression profiles were analyzed in the soybean root subjected to salt stress and dehydration (Wang et al., 2020); the responses of *CsGRAS* genes subjected to salt, drought, cold, and heat treatments were also assessed (Wang et al., 2018). These observations indicate that they probably play a vital role in improving the defensive ability of the plant against abiotic stress. Meanwhile, the RT-qPCR results shown in **Figure 4** and **Supplementary Figure 2**, suggest that the expression levels of 6 *BpGRASs* were upregulated under salt stress; this was consistent with the results of *cis*-acting element analysis (**Figure 6**). Similar results were described in other reports. For example, 6 *GmGRAS* genes, the promoters of which included MYC and GT-1, exhibited notably higher expression levels under drought and salt stress conditions (Wang et al., 2020). *SbGRAS03* was significantly induced by NaCl treatment at the seedling stage, and its expression level was the highest at 2 h (Fan et al., 2021b). Therefore, we hypothesized that these *BpGRASs* may participate in response to abiotic stress.

Several reports have shown that GRAS TFs are involved in the abiotic stress response (Grimplet et al., 2016; Yang et al., 2018; Zhang et al., 2020; Liu et al., 2021a; Liu et al., 2021b). High-efficiency transient transformation could enable us to explore expression patterns and stress resistance in a more effective manner (Ji et al., 2014). To analyze the molecular function of *BpGRASs* in the development of resistance to abiotic stress, 6 transiently transformed plants in which *BpGRASs* were overexpressed and inhibited were used, along with control plants (Control). Besides, *BpGRAS34* was randomly selected for stable transformation of birch and two stable transgenic lines (*OE34-5* and *OE34-7*) were successfully obtained by RT-qPCR for further identification (**Supplementary Figure 3**).

Both of transiently and stably transformed birch plants of overexpression of *BpGRASs* could decrease the extent of cell death, electrolyte leakage, and MDA content under salt stress (**Figures 7** and **8B–D**). It has been uniformly reported that the MDA content, a sign of oxidative damage, was measured, confirming that *BrLAS* overexpression conferred drought resistance in transgenic plants (Li et al., 2018). In addition, we found that overexpression of *BpGRASs* could reduce excess ROS accumulation in this study (**Figures 8B, E–H**, **Supplementary Figure 4** and **Supplementary Figure 5A, B**), indicating that *BpGRASs* have functions related to ROS scavenging. Similarly, overexpression of *SlGRAS40*, clustered into the *HAM* subfamily, can enhance the ROS scavenging ability under salt and drought stress in tomato plants (Huang et al., 2015 and Liu et al., 2017). Overexpression of *HcSCL13* dramatically enhanced the salt resistance of mature transgenic Arabidopsis, as it resulted in an increase in the POD activity (Zhang et al., 2020). Generally, proline acts not only as an osmotic agent, but also as a radical scavenger. Overexpression of *VaPAT1* led to an increase in the proline content, which was an important factor for enhancing cold, drought, and salt stress tolerance in transgenic Arabidopsis (Yuan et al., 2016). We found that the overexpression of *BpGRASs* activated proline biosynthesis, which resulted in an increase in the proline content (**Figure 8C** and **Supplementary Figure 5C**). In addition, phenotype of *OE34-5* and *OE34-7* also showed overexpression of *BpGRAS34* could enhance tolerance to salt stress in birch (**Figure 8A**), which was similar to overexpression of *HhGRAS14* in Arabidopsis when exposed to NaCl condition (Ni et al., 2022). Therefore, we proceeded not only a systematic analysis of the *GRAS* gene family in birch plants but also the expression and analysis of *BpGRASs* by genetic engineering technology including high-efficiency transient transformation and stable transformation, illustrated that *BpGRASs* may play a positive role in the development of tolerance to salt stress in birch plants. This study will lay the foundation for clarifying the molecular mechanism of GRAS TFs in response to salt stress in birch plants and provide an insight of birch improvement by the method of genetic engineering in future.

## Conclusion

Collectively, a total of 40 BpGRAS proteins were identified from the birch genome and phylogenetically classified into 17 subfamilies in this study. A total of 26 *BpGRASs* induced by salt stress exhibited obvious expression patterns under salt stress. Both of 6 *BpGRASs* and selected *BpGRAS34* enhanced the tolerance to salt stress by decreasing the extent of cell death and strengthening the ROS scavenging capacity in OE plants. These results suggest that *BpGRASs* may effectively enhance the tolerance of transgenic birch plants, when exposed to salt stress. This study laid a foundation for further elucidating the functions of *BpGRAS* members and provides valuable information about the functions of *GRAS* family genes in the development of resistance to abiotic stress in birch plants, which may be beneficial for birch improvement.

## Data availability statement

The datasets presented in this study can be found in online repositories. The names of the repository/repositories and accession number(s) can be found in the article/**Supplementary Material**.

## Author contributions

ZH, ZT, QZ, ZW, RH, and XX performed the experiments. YW and XJ conceived the experiments, and ZH and ZT analyzed the data. ZH and XJ wrote and revised the manuscript. All authors approved the final manuscript.

## Funding

This work was supported by the Liaoning Province Science Foundation (No. 2020-MS-197) and Scientific Research Project of Liaoning Education Department (No. LSNQN201919).

## Acknowledgments

The authors would like to thank TopEdit (www.topeditsci.com) for its linguistic assistance during the preparation of this manuscript.

## Conflict of interest

The authors declare that the research was conducted in the absence of any commercial or financial relationships that could be construed as a potential conflict of interest.

## Publisher’s note

All claims expressed in this article are solely those of the authors and do not necessarily represent those of their affiliated organizations, or those of the publisher, the editors and the reviewers. Any product that may be evaluated in this article, or claim that may be made by its manufacturer, is not guaranteed or endorsed by the publisher.
