# Supplementary material for: Genome-wide identification, expression and salt stress tolerance analysis of the GRAS transcription factor family in Betula platyphylla
Source: Front Plant Sci. 2022 Oct 24;13:1022076. doi: 10.3389/fpls.2022.1022076 (PMC9638169; doi:10.3389/fpls.2022.1022076)
Supplement: Supplementary file 1 [file DataSheet_1.zip › Supplementary figures.docx]

**Supplementary Figure 1**


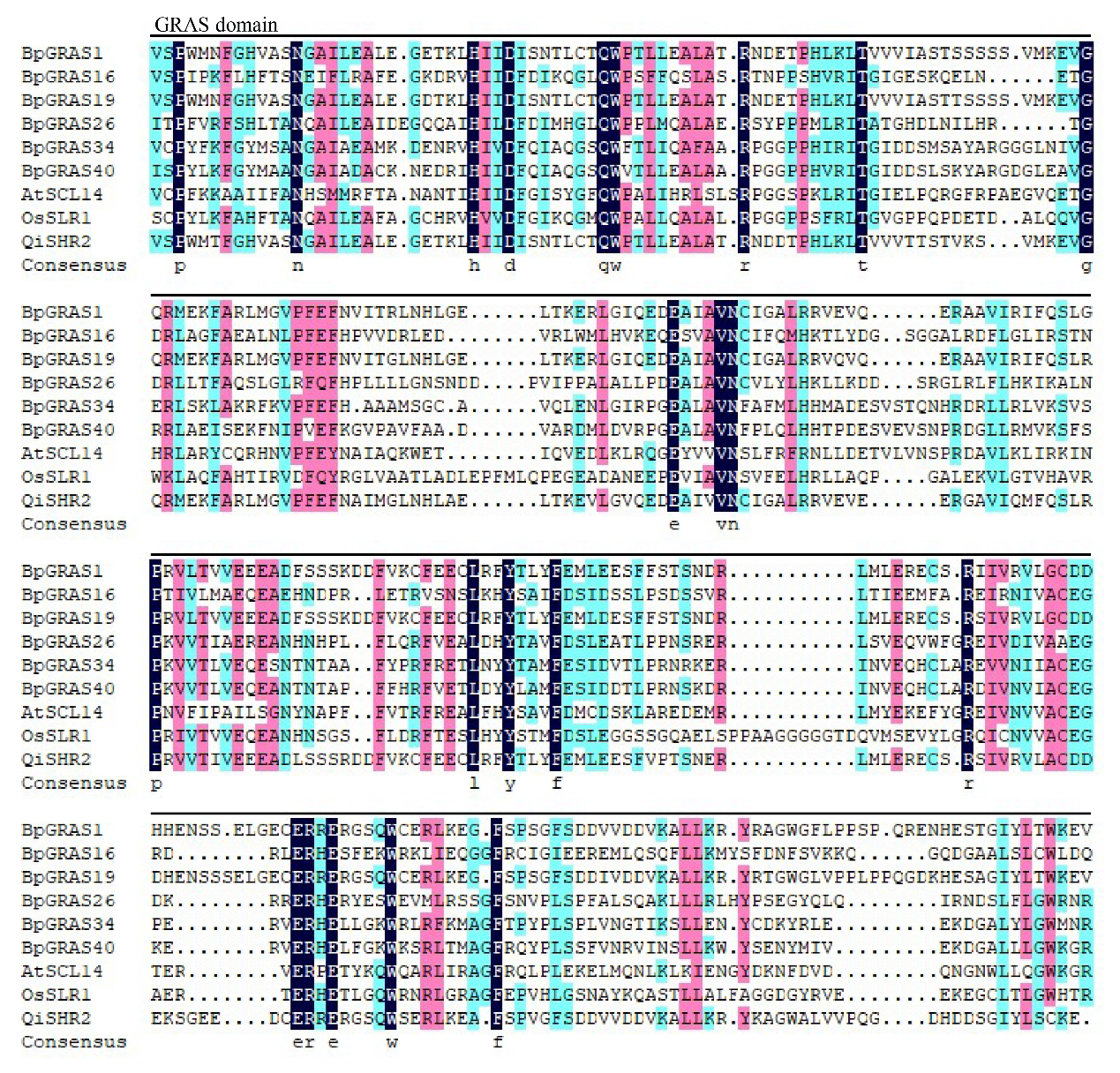


**Supplementary Figure 1. Multiple sequence alignment analysis of 6 BpGRAS proteins obtained from three other plant species.** BpGRAS1: *Betula platyphylla* (MN117546); BpGRAS16: *Betula platyphylla* (MN117547); BpGRAS19: *Betula platyphylla* (MN117548); BpGRAS26: *Betula platyphylla* (MZ062900); BpGRAS34: *Betula platyphylla* (MZ062901); BpGRAS40: *Betula platyphylla* (MZ062902); AtSCL14: *Arabidopsis thaliana* (AT1G07530); OsSLR1: *Oryza sativa* (BGIOSGA013420-PA); and QiSHR2: *Quercus ilex* (AIO08307.1).

**Supplementary Figure 2**


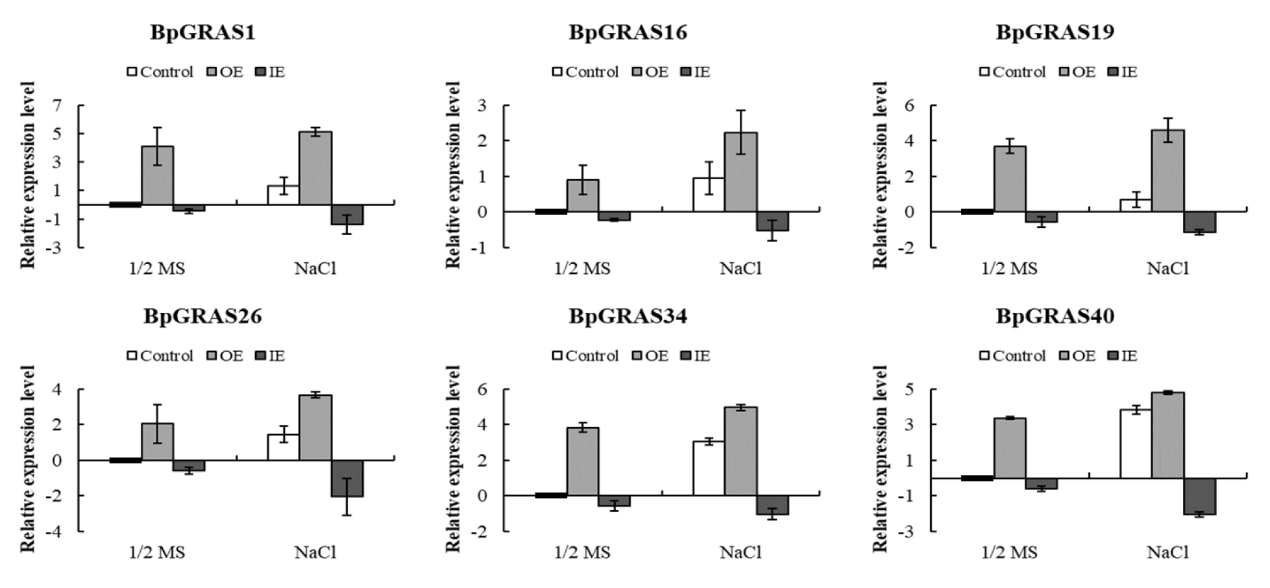


**Supplementary Figure 2. Expression of 6 *BpGRASs* in control, OE, and IE birch plants.** Plants were cultured in 1/2 MS medium (normal conditions) or 1/2 MS medium containing 150 mM NaCl for 24 h to determine the expression level of *BpGRASs*. The expression level of *BpGRASs* after 48 h of transformation in control plants treated with 1/2 MS was regarded as a calibrator (designated as 0), and used to standardize the expression of *BpGRASs* in other plants. Control: birch plants transformed with empty pROKII; OE: birch plants exhibiting overexpression of *BpGRAS*; IE: birch plants exhibiting inhibited expression of *BpGRAS*. Three independent experiments were performed, and data are means ± SD from the three experiments. **Supplementary Figure 3**


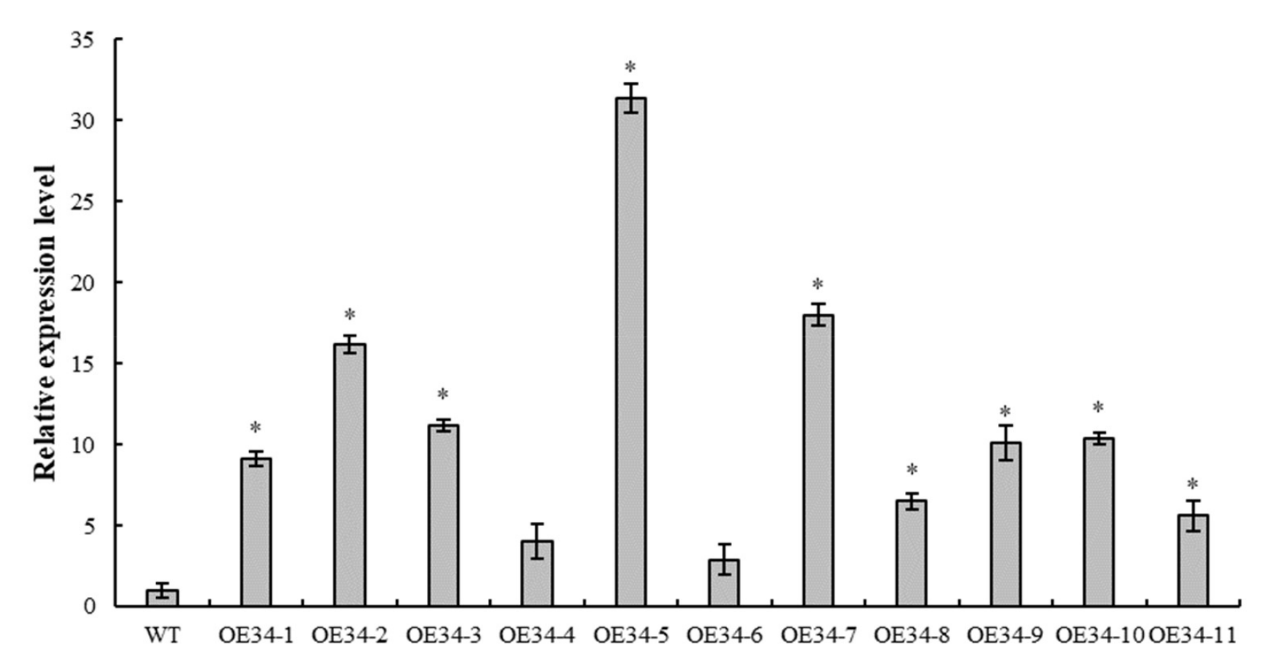


**Supplementary Figure 3. Expression levels of stable transgenic lines of *BpGRAS34*** **overexpression.** WT birch plants was regarded as a calibrator (designated as 1) and used to standardize the expression of stable transgenic lines of *BpGRAS34* overexpression. Asterisk indicates a significant (^*^P < 0.05, t test) difference compared with the WT plants. Three independent experiments were performed, and data are means ± SD from the three experiments. **Supplementary Figure 4**


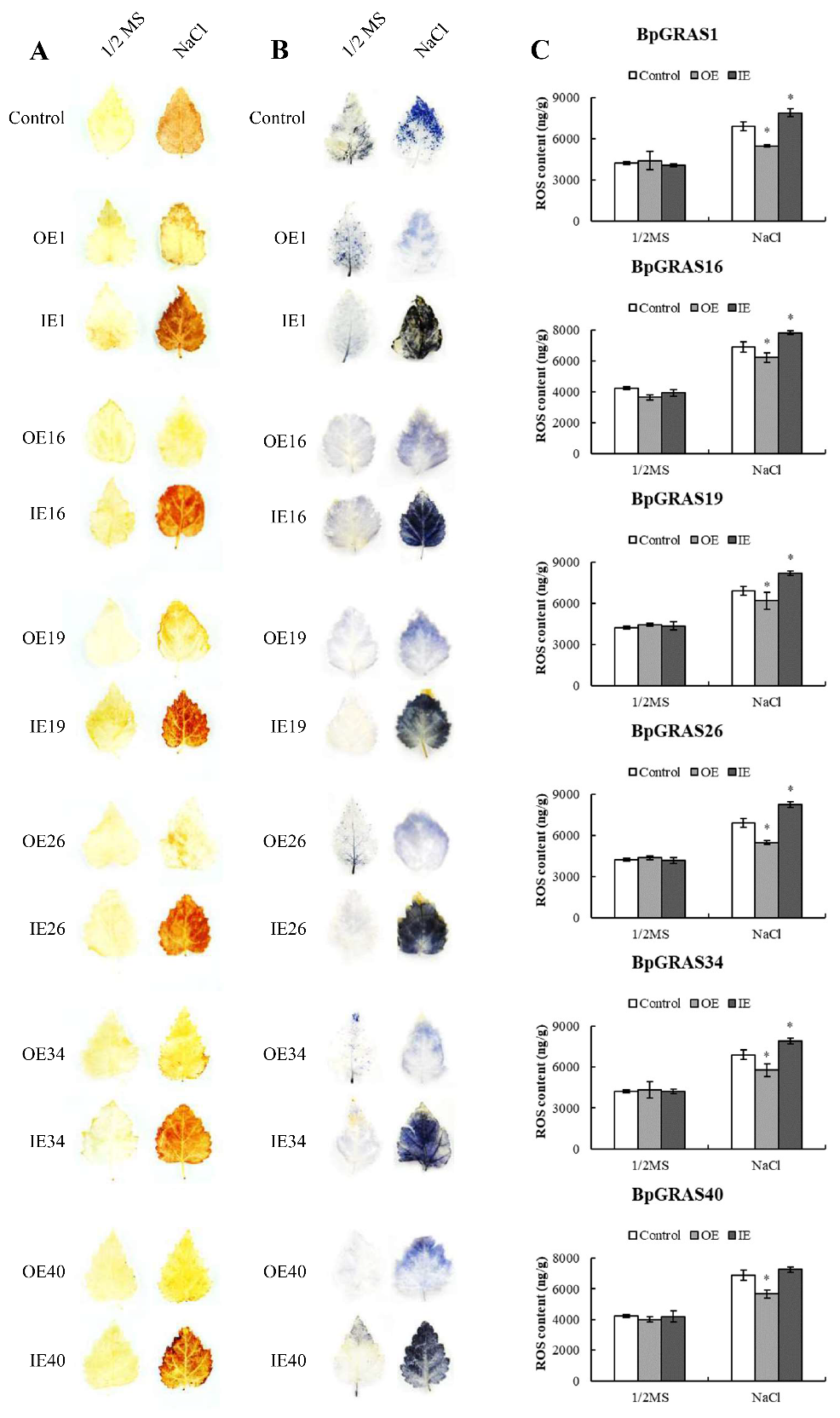


**Supplementary Figure 4. Analysis of ROS accumulation in OE, IE, and control plants.** **(A)**, (**B)** Birch plants treated with 150 mM NaCl and stained with DAB to visualize H_2_O_2_ **(A)**, or stained with NBT to visualize $\text{O}_{\text{2}}^{\text{- .}}$ **(B)**. **(C)** Measurement of ROS accumulation in birch plants. Control: birch plants transformed with empty pROKII; OE: birch plants exhibiting overexpression of *BpGRAS*; IE: birch plants exhibiting inhibited expression of *BpGRAS*. * significant (P < 0.05) difference was observed compared with control plants. Three independent experiments were performed, and data are means ± SD from the three experiments. **Supplementary Figure 5**


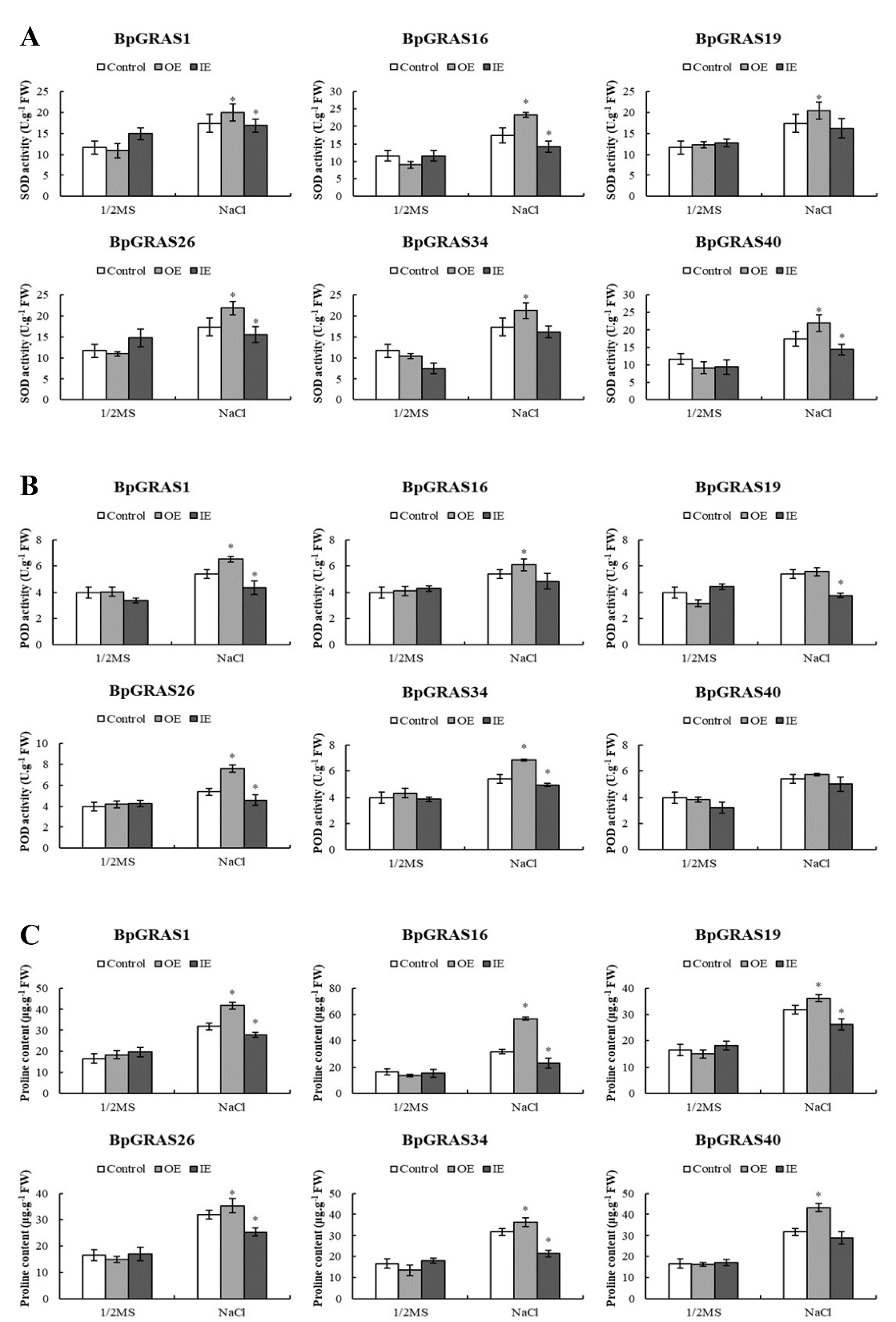


**Supplementary Figure 5**. **Analysis of the POD and SOD activities and proline levels in OE and control plants. (A), (B)** Analysis of SOD **(A)** and POD **(B)** activities in OE and control plants in response to NaCl (150 mM) stress treatment. **(C)** Analysis of proline levels. * significant (P < 0.05) difference was observed compared with control plants. Three independent experiments were performed, and data are means ± SD from the three experiments. Control: birch plants transformed with empty pROKII; OE: birch plants exhibiting overexpression of *BpGRAS*; IE: birch plants exhibiting inhibited expression of *BpGRAS*.
