# Supplementary material for: Genome-wide identification, expression and salt stress tolerance analysis of the GRAS transcription factor family in Betula platyphylla
Source: Front Plant Sci. 2022 Oct 24;13:1022076. doi: 10.3389/fpls.2022.1022076 (PMC9638169; doi:10.3389/fpls.2022.1022076)
Supplement: Supplementary file 1 [file DataSheet_1.zip › Supplementary Tables.docx]

Supplementary Table 1. The primers used for RT-qPCR.

| **Genes** | **Forward primers (5'-3')** | **Reverse primers (5'-3')** |
| --- | --- | --- |
| *actin* | TGAGAAGAGCTATGAGTTGC | GTAGATCCACCACTAAGCAC |
| *β-tubulin* | GTTAGCGAGCAGTTTACAGC | ACCAACAACCTCTTCTTCTT |
| *BpGRAS1* | CAACAAGTAATGACAGATTG | TCATCCACAACATCATCACT |
| *BpGRAS4* | CGTTGAGATTGTTGACTTTG | AGCAACTGCTGCTCCACCAT |
| *BpGRAS5* | GCAGTCAGAATCATCGTGAC | AGTGCTGCTCAACGTTGATC |
| *BpGRAS8* | CTGTTCAGCGAGTGGTTTAC | TACAATGGCTTGGATGCCAG |
| *BpGRAS11* | TGCTCGGATCTCCGAACCAT | TCACATTCTCAATCTTCCAG |
| *BpGRAS12* | TGAAGAGTGTTTGAGATGGT | ACTTCGTCACTGAACGAGAC |
| *BpGRAS13* | CAAGATTGTGGTCTCTTTGG | GTAGCGACCCAGCACGATT |
| *BpGRAS14* | TGTTCGAGTTGCACAAGCTT | GACATCATCTTGTCGTTGCT |
| *BpGRAS16* | GATTGGAAACAAGAGTATCC | CTTGCTCTATCAACTTCCT |
| *BpGRAS18* | GCATTTGGTGCATTACCAAG | TCAGTCTCCTACCTTTCAAC |
| *BpGRAS19* | ATGACAGATTGATGTTGGAG | CACAATATCATCACTAAACC |
| *BpGRAS20* | GTGAGAATCAAGAGAGGTTG | CATCAGCTCTTGGTCCAATG |
| *BpGRAS21* | GGTTGAAGAGACGAGTCCAC | CTACAACATTCATAGCCT |
| *BpGRAS22* | GATTCAAAGAAGCTCTCCAC | TGCCACTGCTTGTACGTCT |
| *BpGRAS23* | CCTTCTTTGTCACACGATTC | CTGCCACTGCTTGTACTTCT |
| *BpGRAS26* | TACACAAACTCCTCAAGGAC | AACTTGCTCCACACTGAGC |
| *BpGRAS29* | ATTCGACCATGTTCGATTCG | TTGGATCCTAAGTGCAATGG |
| *BpGRAS30* | GTGAACTCCGTTTTCGAGCT | GAATCGAACAGGCTGGAAT |
| *BpGRAS31* | AGTCGCTGAACCCAGAAATC | CCAGCAATAGTCTTTCCACT |
| *BpGRAS32* | ACCTCTTTCCTCAACGATCT | ACAGTCTCGATCTTCTTGAC |
| *BpGRAS33* | ATCTCTTGGAGTCTCTGGAT | TGAAGTTGCTGAATGTTACC |
| *BpGRAS34* | TACTGCTGCATTCTATCCAC | ACTTACCAAGAAGCTCATG |
| *BpGRAS36* | ACCTCCGAATCACTGCCAT | AGCATACAATTGATCACAAG |
| *BpGRAS37* | TTGTTGGGTCACTCCACTAC | CAATCCTCCAATGCTTGTAC |
| *BpGRAS38* | CCTTTCTTCCATAGGTTTGT | TCAATCTCGACTTCCATTTG |
| *BpGRAS40* | TTGAGTCGATTGATGACACC | AAGAGCTCAGTGGATATTG |

Supplementary Table 2. The primers used for construction of vectors.

| **Primer names** | **Primer sequences (5’-3’)** |
| --- | --- |
| 35S:BpGRAS1F | CTCTAGAGGATCCCCATGAAATTGACGCTTTGCAAAG |
| 35S:BpGRAS1R | TCGAGCTCGGTACCCTCATGGTTTCCATGCTGAAGC |
| 35S:BpGRAS16F | CTCTAGAGGATCCCCATGTTGGCTGGGTGTTCTAGT |
| 35S:BpGRAS16R | TCGAGCTCGGTACCCTCAACTTGGGTGAGAATAAGAG |
| 35S:BpGRAS19F | CTCTAGAGGATCCCCATGGACACAACCCTTTTCACT |
| 35S:BpGRAS19R | TCGAGCTCGGTACCCTCATGGTTTCCATGCTGAAGC |
| 35S:BpGRAS26F | CTCTAGAGGATCCCCATGTTCATGGCATCATTCAAT |
| 35S:BpGRAS26R | TCGAGCTCGGTACCCTCAGTGCCAGGAAGATACTGAG |
| 35S:BpGRAS34F | CTCTAGAGGATCCCCATGTCCAACGGATTGTACTAT |
| 35S:BpGRAS34R | TCGAGCTCGGTACCCTCACTTCCATGCACAAGCAG |
| 35S:BpGRAS40F | CTCTAGAGGATCCCCATGGAGCCTGATGATGATGAAG |
| 35S:BpGRAS40R | TCGAGCTCGGTACCCTCATTGCCAGGCAGAAGCCGAT |
| pFGC: BpGRAS1-CisF | CCCATGGCACACACATGGACATAACTC |
| pFGC: BpGRAS1-CisR | AGGCGCGCCTCTTGCACACTCGTTTAG |
| pFGC: BpGRAS1-AntiF | CTCTAGACACACACATGGACATAACTC |
| pFGC: BpGRAS1-AntiR | CGCGGATCCTCTTGCACACTCGTTTAG |
| pFGC: BpGRAS16-CisF | CCCATGGTGCCTTCAATGAGCACACAGAGATTG |
| pFGC: BpGRAS16-CisR | TTGGCGCGCCGACCCCTGTTCCGCTAACCTCTT |
| pFGC: BpGRAS16-AntiF | CTCTAGATGCCTTCAATGAGCACACAGAGATTG |
| pFGC: BpGRAS16-AntiR | CGGATCCGACCCCTGTTCCGCTAACCTCTT |
| pFGC: BpGRAS19-CisF | CCCATGGCCACTGCAACCAGCAGAT |
| pFGC: BpGRAS19-CisR | TTGGCGCGCCTTAGCATCCATAGAAGG |
| pFGC: BpGRAS19-AntiF | GCTCTAGACCACTGCAACCAGCAGATT |
| pFGC: BpGRAS19-AntiR | CGGATCCTTAGCATCCATAGAAGG |
| pFGC: BpGRAS26-CisF | CCCATGGGGACCCCAGCTCAGATCTGC |
| pFGC: BpGRAS26-CisR | TTGGCGCGCCTCCGGCTGGTCTGTGTCTGC |
| pFGC: BpGRAS26-AntiF | CTCTAGAGGACCCCAGCTCAGATCTGC |
| pFGC: BpGRAS26-AntiR | CGGATCCTCCGGCTGGTCTGTGTCTGC |
| pFGC: BpGRAS34-CisF | CCCATGGCAGCTATGCTACAATGATAG |
| pFGC: BpGRAS34-CisR | TTGGCGCGCCTCCACATATAGAAGAGCCAT |
| pFGC: BpGRAS34-AntiF | CTCTAGACAGCTATGCTACAATGATAG |
| pFGC: BpGRAS34-AntiR | CGGATCCTCCACATATAGAAGAGCCAT |
| pFGC: BpGRAS40-CisF | CATGCCATGGCAGAGACACCGGGCCAG |
| pFGC: BpGRAS40-CisR | AGGCGCGCCTAAATGGTTGCGCCCGATTCC |
| pFGC: BpGRAS40-AntiF | CTCTAGACAGAGACACCGGGCCAG |
| pFGC: BpGRAS40-AntiR | CGGATCCTAAATGGTTGCGCCCGATTC |
